# Exploiting the Electronic Tuneability of Carboranes as Supports for Frustrated Lewis Pairs

**DOI:** 10.3390/molecules23123099

**Published:** 2018-11-27

**Authors:** Amanda Benton, Zachariah Copeland, Stephen M. Mansell, Georgina M. Rosair, Alan J. Welch

**Affiliations:** Institute of Chemical Sciences, School of Engineering & Physical Sciences, Heriot-Watt University, Edinburgh Scotland EH14 4AS, UK; ab437@hw.ac.uk (A.B.); z1309c@yahoo.co.uk (Z.C.); g.m.rosair@hw.ac.uk (G.M.R.)

**Keywords:** carborane, phosphine, frustrated Lewis pair, catalysis

## Abstract

The first example of a carborane with a catecholborolyl substituent, [1-Bcat-2-Ph-*closo*-1,2-C_2_B_10_H_10_] (**1**), has been prepared and characterized and shown to act as the Lewis acid component of an intermolecular frustrated Lewis pair in catalyzing a Michael addition. In combination with B(C_6_F_5_)_3_ the *C*-carboranylphosphine [1-PPh_2_-*closo*-1,2-C_2_B_10_H_11_] (**IVa**) is found to be comparable with PPh_2_(C_6_F_5_) in its ability to catalyze hydrosilylation, whilst the more strongly basic *B*-carboranylphosphine [9-PPh_2_-*closo*-1,7-C_2_B_10_H_11_] (**V**) is less effective and the very weakly basic species [μ-2,2′-PPh-{1-(1′-1′,2′-*closo*-C_2_B_10_H_10_)-1,2-*closo*-C_2_B_10_H_10_}] (**IX**) is completely ineffective. Base strengths are rank-ordered via measurement of the ^1^*J* ^31^P-^77^Se coupling constants of the phosphineselenides [1-SePPh_2_-*closo*-1,2-C_2_B_10_H_11_] (**2**), [9-SePPh_2_-*closo*-1,7-C_2_B_10_H_11_] (**3**), and [SePPh_2_(C_6_F_5_)] (**4**).

## 1. Introduction

The recognition by Stephan and co-workers, little more than a decade ago [1], that H_2_ could be reversibly activated using sterically-encumbered main group Lewis acid (LA)/Lewis base (LB) pairs has given rise to the burgeoning field of frustrated Lewis pair (FLP) chemistry [2,3,4,5,6,7,8]. FLPs can co-exist on the same molecule (intramolecular FLPs) or be on different molecules (intermolecular FLPs). Since its inception, the breadth of FLP chemistry has expanded considerably, and now, as summarized in a recent review [9], impacts upon small-molecule activation, organic chemistry, radical chemistry, transition-metal chemistry, enzyme models, polymers and materials, and surface chemistry.

Carborane chemistry is a well-established and wide-ranging field with thousands of derivatives known and a huge number of diverse applications now established for carborane-containing species [10]. Thus far, however, FLP chemistry and carborane chemistry have not intersected, in spite of the fact that the carborane scaffold offers a number of unique advantages for potential FLPs including high chemical and thermal stability, the ability to act as an electron-donating or electron-accepting support dependent on the vertex substituted (with no significant difference in the steric bulk of the carborane) [11,12,13], and further tuneability of electronic (and steric) FLP properties through isomerization, cage derivatization, or substitution [10].

We now report the first examples of intermolecular carborane-supported FLP chemistry, through (i) the synthesis of a catecholborolyl (Bcat) carborane (the LA) and its catalytic activation of a Michael addition reaction in combination with PPh_3_, and (ii) a comparison of *C*- and *B*-carboranylphosphines (the LB) in combination with B(C_6_F_5_)_3_ to catalyze a hydrosilylation reaction, demonstrating the unique advantage of carborane scaffolds in tuning the strength of FLP components.

## 2. Results and Discussion

### 2.1. Synthesis and Characterization of Compound 1

Following deprotonation of [1-Ph-*closo*-1,2-C_2_B_10_H_11_] in tetrahydrofuran (THF) by *^n^*BuLi and subsequent exchange of solvent for toluene, 2-*Br*-1,3,2-benzodioxaborole (BcatBr) in toluene was added and the reaction mixture heated to reflux overnight. The solvent was removed and the product extracted into petroleum ether. Unreacted phenylcarborane was removed via vacuum sublimation, leaving the product [1-Bcat-2-Ph-*closo*-1,2-C_2_B_10_H_10_] (**1**) in a 45% isolated yield. As far as we are aware, compound **1** is the first example of a carborane with a catecholborolyl substituent. *C*-substituted pinacolborolyl (Bpin) carboranes are known (see, e.g., References [14,15]) (there is one recent report of a *B*-substituted pinacolborolyl carborane [16]) and several *C*-substituted diazaborolyl carboranes have been prepared, e.g., References [17,18].

Compound **1** is a moderately air-sensitive, but significantly moisture-sensitive, colorless solid, initially characterized using elemental analysis, mass spectrometry, and by ^1^H and ^11^B nuclear magnetic resonance (NMR) spectroscopies. The latter features the resonance due to the catecholborolyl B atom at δ 28.8 ppm in C_6_D_6_, easily identified by its high frequency chemical shift and lack of ^1^H coupling. The carboranyl boron resonances in the ^11^B{^1^H} NMR spectrum appear with relative integrals 1:1:2:2:2:2 from high frequency to low frequency suggest a molecule with time-averaged *C*_s_ molecular symmetry in solution.

**Figure 1:**
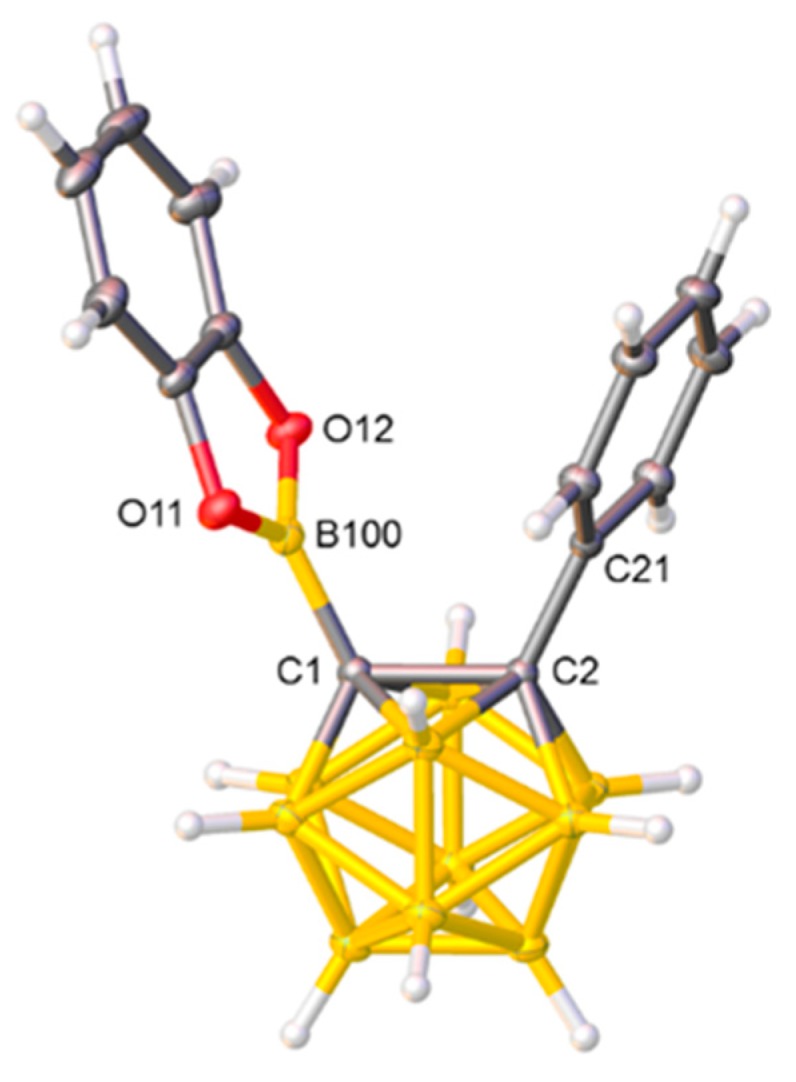
Molecular structure of compound **1**. Selected interatomic distances (Å): C1−C2 1.6840(15), C1−B100 1.5703(16), B100−O11 1.3744(14), B100−O12 1.3739(15), and C2−C21 1.5083(14).

Ultimately compound **1** was unambiguously characterized crystallographically. Single crystals suitable for an X-ray diffraction study were grown by cooling a solution of **1** in C_6_H_5_F and a perspective view of a single molecule is shown in Figure 1. The *C*_s_ symmetry in solution was not retained in the solid state as the Ph and Bcat units were twisted by ≈19° in a conrotatory manner with respect to the least-squares plane through atoms B100C1C2C21. The C1−C2 distance in **1**, 1.6840(15) Å, sits within the range of such distances in related compounds (see, e.g., References [17,18]), whilst the C1−B100 distance, 1.5703(16) Å, was comparable to that (1.565(4) Å) in [1-Bpin-2,3,4,5,6,7,8,9,10,11-Me_10_-12-(4′-Br-C_6_H_4_)-*closo*-1-CB_11_]^−^ [14] but significantly shorter than that (1.6046(19) Å) in [1-B(OMe)_2_-2-*^i^*Pr-*closo*-1,2-C_2_B_10_H_10_] [15], the only other compounds with {BO_2_} fragments bonded to a carborane C atom to have been structurally characterized.

### 2.2. Carborane-Supported Components of Intermolecular FLPs to Catalyze Michael Addition

**Scheme 1:**
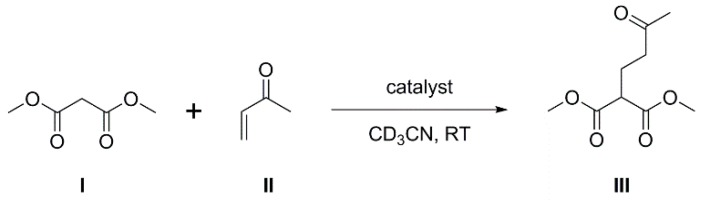
Michael addition of dimethyl malonate and 3-buten-2-one producing dimethyl 2-(3-oxobutyl) malonate.

The steric bulk and electron-withdrawing nature of the phenylcarborane fragment results in B100 being both sterically-encumbered and highly Lewis acidic, and consequently compound **1** is an ideal potential LA component of an intermolecular FLP. To investigate this, we have studied the involvement of **1** as co-catalyst in the Michael addition of dimethyl malonate (**I**) to 3-buten-2-one (**II**) to afford dimethyl 2-(3-oxobutyl) malonate (**III**), a classic Michael addition reaction (Scheme 1). Such reactions are known to be catalyzed by phosphines alone [19], but enhanced rates have been observed if a species with the potential to act as a Lewis acid is also present because the LA and LB form an FLP (see, e.g., References [20,21,22]).

In the presence of 10 mol% PPh_3_ in CD_3_CN at room temperature, a 1:1 mixture of **I** and **II** affords **III** in 43% yield after 6 h and 64% yield after 24 h (entries 1 and 2, Table 1). No reaction was observed in the absence of a catalyst nor in the presence of only the Bcat carborane **1**. When, however, an intermolecular FLP of 10 mol% **1** and 10 mol% PPh_3_ was used as a catalyst, the yield of **III** was 56% after 6 h and 76% after 24 h (entries 3 and 4). This demonstrates the co-operative nature of the two components, with the enhancement in catalysis fully consistent with them acting as a frustrated Lewis pair and represents the first time that a carborane-containing species has been used as a component of an FLP. The results are at least as good as those obtained for the same reaction using either PhBpin/PPh_3_ as an intermolecular FLP or 1-Bpin-2-PPh_2_-C_6_H_4_ as an intramolecular FLP [22].

**Scheme 2:**
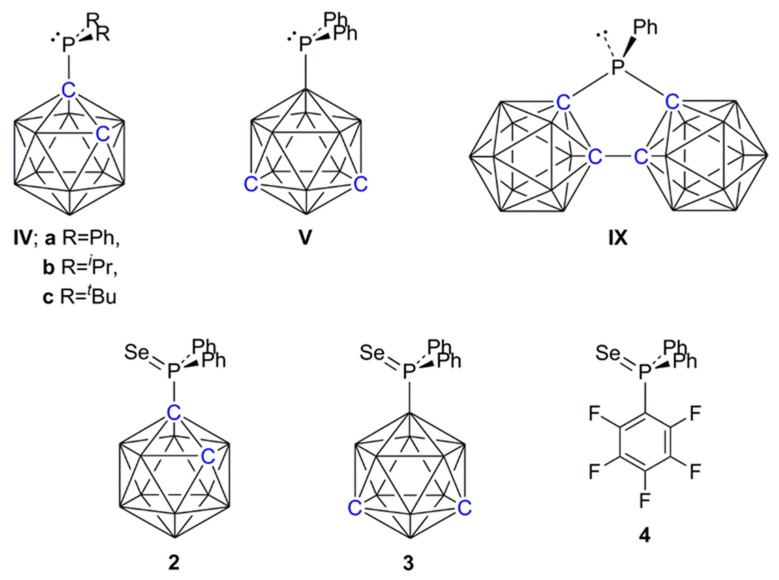
Line diagrams of key species.

An interesting alternative to using PPh_3_ as a catalyst for this Michael addition reaction would be to have the LB functionality on a carborane cage. However, the *C*-carboranylphosphines [1-PR_2_-*closo*-1,2-C_2_B_10_H_11_] [R = Ph (**IVa**), *^i^*Pr (**IVb**), *^t^*Bu (**IVc**) (Scheme 2)] were all inactive in catalyzing the reaction, presumably a consequence of their relatively low basicity because of the strong electron-acceptor property of the carborane when substituted at C. In contrast, it is well established that a carborane substituted at B is electron-releasing [11,12,13], and we therefore prepared the *B*-substituted carboranylphosphine [9-PPh_2_-*closo*-1,7-C_2_B_10_H_11_] (**V**) [13] and tested it as the single catalyst for Michael addition, finding it to be significantly more effective than PPh_3_ (Table 1, entries 5 and 6). Note that **IVa** and **V** are related as simple positional isomers. Replacing a Ph group in PPh_3_ with a *C*-bound carborane cage (affording **IVa**) reduces the basicity of the phosphine and switches off the Michael addition reaction, whilst replacing a Ph in PPh_3_ with a *B*-bound carborane cage (affording **V**) enhances this particular catalysis. This clearly demonstrates the potential of electronically-flexible carborane scaffolds in controlling the properties of substituents and so optimizing catalysis.

### 2.3. Carborane-Supported Components of Intermolecular FLPs to Catalyze Hydrosilylation

**Scheme 3:**
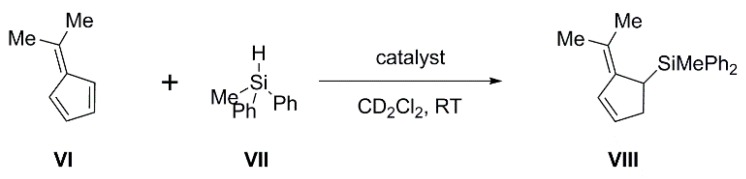
Hydrosilylation of dimethylfulvene.

Although the *C*-substituted carboranylphosphines **IV** are too weakly basic for Michael addition, they can be, as part of an FLP, highly effective in the hydrosilylation of dimethylfulvene (Scheme 3). In combination with B(C_6_F_5_)_3_, the weak Lewis base PPh_2_(C_6_F_5_) has been shown by Paradies and co-workers to catalyze this reaction effectively, whilst the strong Lewis base P*^t^*Bu_3_ has no activity (it is assumed that this is due to the formation of the silylium salt [*^t^*Bu_3_P-SiPh_2_Me][HB(C_6_F_5_)_3_]) [23]. This is therefore an ideal reaction in which to study the effect of controlling the basicity of the Lewis base component of the FLP and we have investigated the effectiveness of carborane supports in affording that control.

In Table 2, the intermolecular FLPs formed by B(C_6_F_5_)_3_ with PPh_2_(C_6_F_5_), **IVa** and **V** are compared for their efficiency in catalyzing this reaction. The FLP B(C_6_F_5_)_3_/**IVa** was fully comparable with B(C_6_F_5_)_3_/PPh_2_(C_6_F_5_) in efficiency, both combinations producing nearly 90% product yield after only 11–12 min (entries 1 and 2), whilst the FLP from B(C_6_F_5_)_3_ with the more strongly basic **V** was inferior to both, affording the product only 80% yield after more than twice the time (entry 3). We also investigated the effect on the reaction of using the FLP formed from B(C_6_F_5_)_3_ and the very weakly basic bis(carboranyl)phosphine [μ-2,2′-PPh-{1-(1′-1′,2′-*closo*-C_2_B_10_H_10_)-1,2-*closo*-C_2_B_10_H_10_}] (**IX**) [24] (Scheme 2). In this case significant amounts of oligomerized product were immediately observed (entry 4), consistent with the results obtained by Paradies when no base was used [23]. These results clearly establish that this reaction was very sensitive to the strength of the Lewis base component; if the base is too strong (e.g., P*^t^*Bu_3_) there is no catalysis [23], whilst if it is too weak (e.g., **IX**), the base plays no part in the chemistry and the Lewis acid catalyzes oligomerization. Between these two extremes, the base acts as an FLP with the B(C_6_F_5_)_3_ Lewis acid, which catalyzes hydrosilylation, with weaker bases performing somewhat better.

### 2.4. Synthesis and Characterization of Phosphineselenides 2, 3 and 4

In an attempt to understand the relative efficiencies of the phosphines PPh_3_, **IVa**, **V**, **IX**, and PPh_2_(C_6_F_5_) as stand-alone Lewis bases or as components of FLPs, we have attempted to rank their basicities via formation of the appropriate selenide. This is because it is well established that, in the absence of significant intra- or intermolecular H-bonding contacts, p*K*_b_ of phosphines correlates almost linearly with the magnitude of the ^1^*J* ^31^P-^77^Se coupling constants of the corresponding selenide (see, e.g., References [25,26].

The selenides [1-SePPh_2_-*closo*-1,2-C_2_B_10_H_11_] (**2**, derived from **IVa**), [9-SePPh_2_-*closo*-1,7-C_2_B_10_H_11_] (**3**, derived from **V**) and [SePPh_2_(C_6_F_5_)] (**4**) were prepared in good yields using the straightforward procedure of heating to reflux the appropriate phosphine and excess Se in toluene. All three pale or colorless compounds were crystalline solids that were initially characterized using elemental analysis, mass spectrometry, and ^1^H and ^31^P{^1^H} NMR spectroscopies, plus ^11^B{^1^H} (for **2** and **3**), ^77^Se (for **3**), and ^19^F{^1^H} (for **4**) NMR studies.

**Figure 2:**
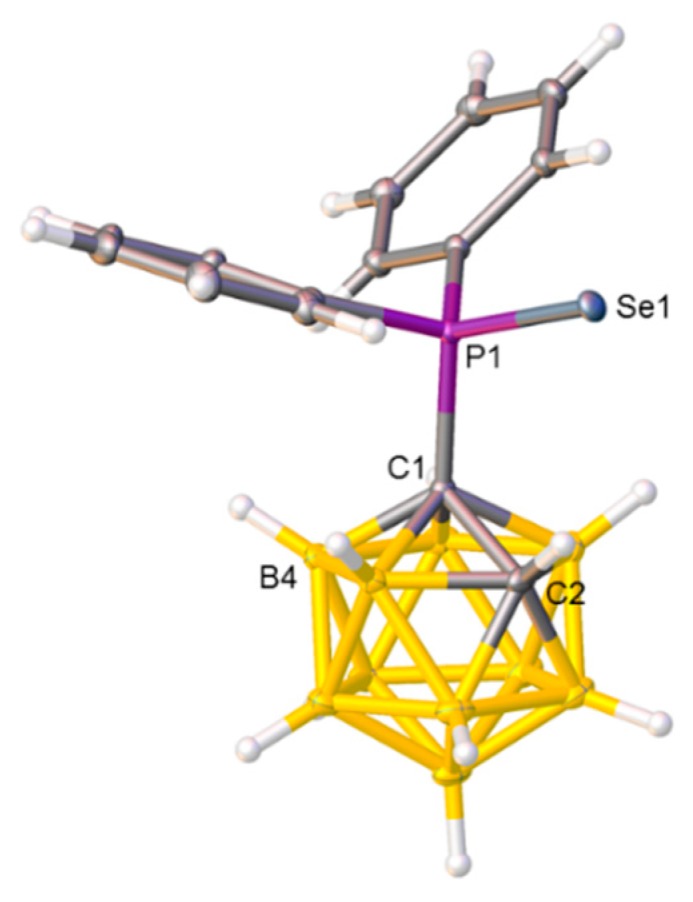
Molecular structure of compound **2**. Selected interatomic distances (Å): C1−C2 1.6511(14), C1−P1 1.8869(10), and P1−Se1 2.1037(3).

**Figure 3:**
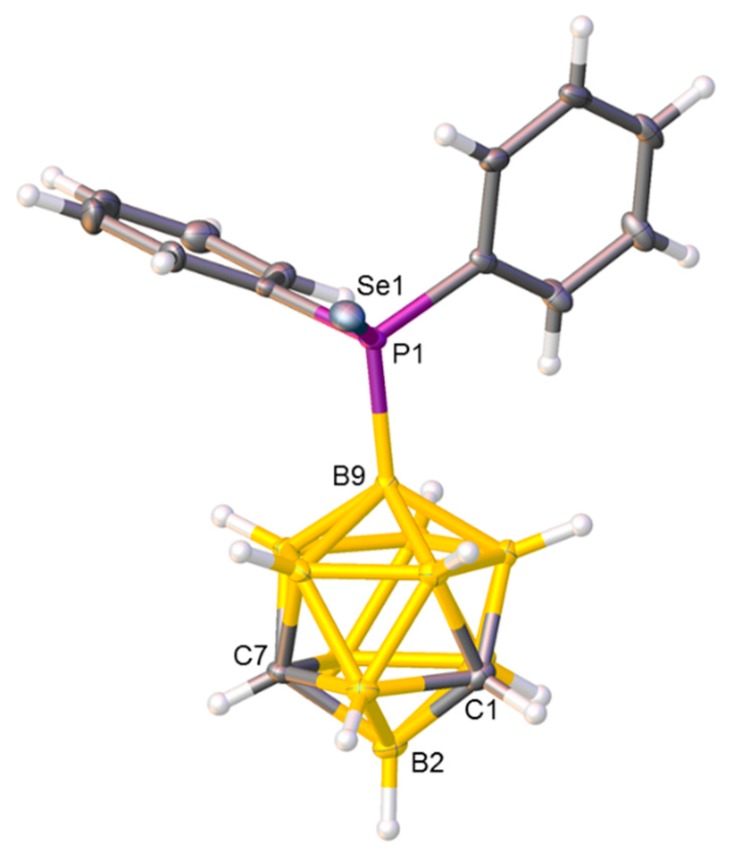
Molecular structure of compound **3**. Selected interatomic distances (Å): B9−P1 1.937(2) and P1−Se1 2.1196(5).

**Figure 4:**
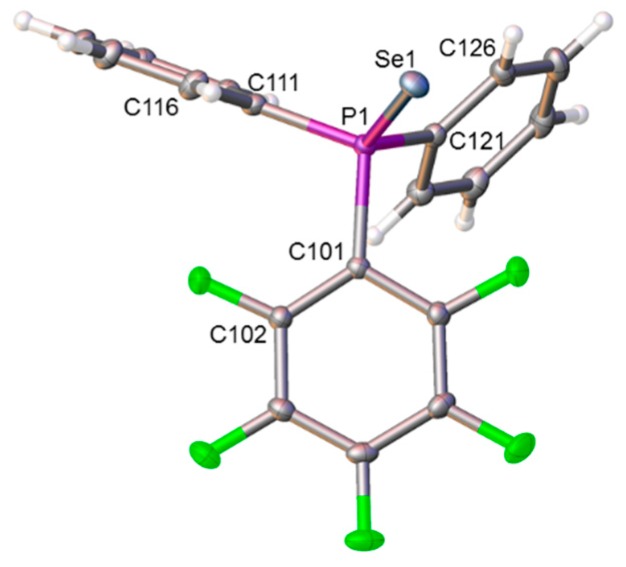
Molecular structure of compound **4**. Selected interatomic distances (Å): P1−Se1 2.1047(3), P1−C101 1.8346(11), P1−C111 1.8141(12), and P1−C121 1.8080(12).

Compounds **2**, **3**, and **4** were also studied crystallographically, and perspective views of single molecules together with key molecular parameters are presented in Figure 2, Figure 3, and Figure 4, respectively. In **2**, there appears to be a preferred orientation of the {SePPh_2_} fragment relative to the carborane cage with the torsion angle C2−C1−P−Se only 5.73(7)°, allowing the Se atom and the relatively protonic H bound to C2 to approach to within 2.752(17) Å, substantially less than the sum of their van der Waals radii of ≈3.10 Å [27].

In all cases, the ^31^P{^1^H} NMR shifts of the selenides **2**, **3**, and **4** were observed at significantly higher frequency than those of the precursor phosphine (δ 50.7 ppm vs 25.3 ppm for **2**, +3.8 ppm vs −48.2 ppm for **3**, +20.4 ppm vs −25.0 ppm for **4**, all comparisons made in the same solvent). Most importantly, ^77^Se satellites reveal ^1^*J* ^31^P-^77^Se coupling constants of 799 Hz for **2**, 704 Hz for **3** (confirmed using the ^77^Se NMR spectrum), and 774 Hz for **4**. SePPh_3_ (^1^*J* ^31^P-^77^Se = 732 Hz) [25] and Se**IX** (^1^*J* ^31^P-^77^Se = 891 Hz) [24] are known species.

Based on the magnitude of these ^1^*J*_PSe_ values, the ranking of the base strengths of the phosphines was **V** (most basic) > PPh_3_ > PPh_2_(C_6_F_5_) > **IVa** > Se**IX** (least basic). This is fully consistent with the observation that **V** acts as the best stand-alone phosphine for catalyzing the Michael addition reaction but is the worst LB component of an FLP with B(C_6_F_5_)_3_ in catalyzing the hydrosilylation reaction, known to be favored by less basic phosphines [23]. Comparison of the coupling constants of **2** and **3** dramatically illustrates the different basicities of carboranylphosphines substituted at C (compound **IVa**, weakly basic) versus those substituted at B (compound **V**, strongly basic). In an alternative description, starting from PPh_3_, notional replacement of one Ph by (C_6_F_5_) reduces the basicity (as expected), whilst notional replacement of Ph by a *C*-carborane cage, forming [1-PPh_2_-*closo*-1,2-C_2_B_10_H_11_] (**IVa**), reduced the basicity even further (*C*-substituted carborane cage is more electron-withdrawing than Ph). On the other hand, notional replacement of one Ph in PPh_3_ by a *B*-carborane cage, affording [9-PPh_2_-*closo*-1,7-C_2_B_10_H_11_] (**V**), *increased* the basicity (*B*-substituted carborane cage is less electron-withdrawing than Ph).

These results confirm that the nature of the site of substitution of a carborane significantly affects the strength of an appended Lewis base and, by extension, an appended Lewis acid. In principle, the acid and/or base strength can be further tuned by varying the size of the carborane, its structural type (closo, neutral; nido, anionic), its isomeric form, the nature of any heteroatoms present (e.g., metal fragment vertices), and the nature of additional substituents at B and/or at C. Thus, carborane scaffolds have the unique potential to offer an exceptional degree of control over the Lewis acid and/or Lewis base strength of appended groups, which is likely to be highly important in constructing useful FLP combinations for a variety of catalytic applications. Future contributions will explore further these possibilities.

## 3. Experimental Section

### 3.1. General Considerations

All experiments were performed, unless otherwise stated, under an atmosphere of dry nitrogen using standard Schlenk or glovebox techniques, with some subsequent manipulations and purifications carried out in air. Tetrahydrofuran (THF) was distilled from sodium/benzophenone, dichloromethane (DCM) from CaH_2_, and toluene and petroleum ether (40–60 °C, petrol) from sodium. All solvents were freeze-pump-thawed three times prior to use. Deuterated solvents were stored over 4 Å molecular sieves. [1-PPh_2_-*closo*-1,2-C_2_B_10_H_11_] (**IVa**) [28], [1-P*^i^*Pr_2_-*closo*-1,2-C_2_B_10_H_11_] (**IVb**) [29], [1-P*^t^*Bu_2_-*closo*-1,2-C_2_B_10_H_11_] (**IVc**) [30], [9-PPh_2_-*closo*-1,7-C_2_B_10_H_11_] (**V**) [13], [μ-2,2′-PPh-{1-(1′-1′,2′-*closo*-C_2_B_10_H_10_)-1,2-*closo*-C_2_B_10_H_10_}] (**IX**) [24], and [1-Ph-*closo*-1,2-C_2_B_10_H_11_] [31,32] were prepared according to the literature. All other reagents were purchased from Sigma Aldrich Ltd. (Gillingham, UK), Fluorochem Ltd. (Hadfield, UK) or Alfa Aesar (Heysham, UK) and used without further purification. NMR spectra were recorded at 298 K using a Bruker AVIII-400 spectrometer (Bruker BioSpin AG, Fallenden, Switzerland), with chemical shifts reported relative to the residual protonated solvent peaks (^1^H) or to external standards (^11^B; BF_3_∙OEt_2_). Elemental analyses were conducted using an Exeter CE-440 elemental analyser (Exeter Analytical Inc., North Chelmsford, MA, USA). Electron ionization mass spectrometry (EIMS) was carried out on a Bruker Microtof II mass spectrometer (Bruker Daltonik GmbH, Bremen, Germany) at the University of Edinburgh.

#### 3.1.1. Synthesis and Characterization of [1-Bcat-2-Ph-*closo*-1,2-C_2_B_10_H_10_] (**1**)

[1-Ph-*closo*-1,2-C_2_B_10_H_11_] (300 mg, 1.36 mmol) was dried under vacuum and dissolved in anhydrous THF (20 mL). The solution was cooled to 0 °C before *^n^*BuLi (1.02 mL, 1.6M, 1.63 mmol) was added dropwise. The colorless solution turned pale pink and was stirred at 0 °C for 0.5 h before being warmed to room temperature, then heated to 65°C for 1 h. The pale-yellow solution was allowed to cool to room temperature and concentrated to dryness. Anhydrous toluene (25 mL) was added. The Schlenk tube was covered in foil and the pale-yellow solution cooled to −78 °C before the addition of a toluene solution of 2-bromo-1,3,2-benzodioxaborole (324 mg, 1.63 mmol), resulting in a purple solution and a blue precipitate. The mixture was heated to reflux overnight. The purple solution was transferred via cannula to a second Schlenk tube along with anhydrous toluene washings (2 × 20 mL) and concentrated to a purple solid. This was extracted with anhydrous petrol (2 × 50 mL) and the soluble materials evaporated to reveal a white solid. Excess phenyl carborane was removed via vacuum sublimation leaving the product [1-Bcat-2-Ph-*closo*-1,2-C_2_B_10_H_10_] (**1**) (205 mg, 0.61 mmol, 45%). C_14_H_19_B_11_O_2_ requires C 49.7, H 5.66; found C 49.2, H 5.66%. ^1^H NMR (400 MHz, C_6_D_6_): δ 7.44–7.41 (m, 2H, C_6_*H*_5_), 6.74–6.71 (m, 1H, C_6_*H*_5_), 6.67–6.61 (m, 4H), 6.50–6.53 (m, 2H). ^11^B{^1^H}[^11^B] NMR (128 MHz, C_6_D_6_): δ 29.0 (s, 1B, *B*cat), 2.4 (s [d, *J*_BH_ = 150 Hz], 1B), −2.4 (s [d, *J*_BH_ = 150 Hz], 1B), −7.3 (2B), −8.3 (2B), −9.7 (2B), −10.7 (2B). EIMS: envelope centered on *m/z* 338.2 (M^+^).

#### 3.1.2. General Synthesis and Characterization of Phosphine Selenides **2**, **3**, and **4**

The phosphine (typically 0.25–0.5 mmol) was dissolved in toluene (typically 10–15 mL) and ≈10 equivalents of elemental selenium were added. Under N_2_, the reagents were heated to reflux overnight. Excess Se was filtered off and washed with DCM. The filtrate and washings were evaporated to afford essentially pure colorless or pale-colored solids.

[1-SePPh_2_-*closo*-1,2-C_2_B_10_H_11_] (**2**) Colorless, 56% yield. C_14_H_21_B_10_PSe requires C 41.3, H 5.20; found C 40.8, H 5.06%. ^1^H NMR (400 MHz, CDCl_3_): δ 8.28–8.22 (m, 4H, C_6_*H*_5_), 7.64–7.52 (m, 6H, C_6_*H*_5_), 4.80 (br. s, 1H, C_cage_*H*). ^11^B{^1^H} NMR (128 MHz, CDCl_3_): δ −0.5 (1B), −2.5 (1B), −7.2 (2B), −11.0 (2B), −12.8 (4B). ^31^P{^1^H} NMR (162.0 MHz, CDCl_3_): δ 50.66 (s + Se satellites, ^1^*J*_PSe_ = 799 Hz). EIMS: envelope centered on *m/z* 407.1 (M^+^).

[9-SePPh_2_-*closo*-1,7-C_2_B_10_H_11_] (**3**) Pale yellow, 71% yield. C_14_H_21_B_10_PSe requires C 41.3, H 5.20; found C 42.2, H 5.35%. ^1^H NMR (400 MHz, CD_2_Cl_2_): δ 8.04–7.98 (m, 4H, C_6_*H*_5_), 7.47–7.43 (m, 6H, C_6_*H*_5_), 3.13 (br. s, 2H, C_cage_*H*). ^11^B{^1^H} NMR (128 MHz, C_6_D_6_): δ −3.4 (1B), −4.7 (1B), −5.9 (2B), −9.9 (1B), −11.9 (1B), −12.7 (2B), −16.3 (2B). ^31^P{^1^H} NMR (162.0 MHz, C_6_D_6_): δ 3.76 (q [^1^*J*_PB_ = 164 Hz] + Se satellites, ^1^*J*_PSe_ = 704 Hz). ^77^Se NMR (76.4 MHz, C_6_D_6_): δ −230.54 (d, ^1^*J*_PSe_ = 704 Hz). EIMS: envelope centered on *m/z* 408.2 (M^+^).

[SePPh_2_(C_6_F_5_)] (**4**) Pale pink, 57% yield. C_18_H_10_F_5_PSe requires C 50.1, H 2.34; found C 50.1, H 2.34%. ^1^H NMR (400 MHz, CDCl_3_): δ 7.99–7.92 (m, 4H, C_6_*H*_5_), 7.58–7.47 (m, 6H, C_6_*H*_5_). ^19^F NMR (376.5 MHz, CDCl_3_): δ −126.9 (s, 2F, C_6_*F*_5_), −147.1 (s, 2F, C_6_*F*_5_), −158.8 (s, 1F, C_6_*F*_5_). ^31^P{^1^H} NMR (162.0 MHz, CDCl_3_): δ 20.42 (s + Se satellites, ^1^*J*_PSe_ = 774 Hz). EIMS: envelope centred on *m/z* 431.9 (M^+^).

### 3.2. Crystallographic Studies

Compound **1**, crystal data: C_14_H_19_B_11_O_2_, *M* = 338.20, monoclinic, *P*2_1_/*c*, *a* = 15.0049(6), *b* = 6.78992(18), *c* = 19.0043(6) Å, β = 112.135(4)°, *U* = 1793.49(11) Å^3^, *Z* = 4, *D*_c_ = 1.253 Mg m^−3^, μ = 0.069 mm^−1^, *F*(000) = 696. 37112 data to θ_max_ = 31.23° were collected at 120.01(10) K on a Rigaku Oxford Diffraction SuperNova diffractometer using Mo-*K*_α_ X-radiation. A total of 5441 unique reflections (*R*_int_ = 0.0424) were used to solve (using SHELXS [33]) and refine (using SHELXL [34]) the structure within the Olex2 [35] package. *R*_1_ = 0.0480, w*R*_2_ = 0.1189 for data with *I* ≥ 2σ(*I*), *S* (all data) = 1.073, *E*_max_, *E*_min_ = 0.38, −0.23 eÅ^−3^, respectively. CCDC 1848620.

Compound **2**, crystal data: C_14_H_21_B_10_PSe, *M* = 407.34, monoclinic, *P*2_1_/*c*, *a* = 8.7241(4), *b* = 25.2149(11), *c* = 9.4588(4) Å, β = 111.615(2)°, *U* = 1934.41(15) Å^3^, *Z* = 4, *D*_c_ = 1.399 Mg m^−3^, μ = 2.018 mm^−1^, *F*(000) = 816. 58543 data to θ_max_ = 34.07° were collected at 100.00(10) K on a Bruker X8 APEXII diffractometer using Mo-*K*_α_ X-radiation. A total of 7897 unique reflections (*R*_int_ = 0.0451) were used to solve and refine the structure, as for compound **1**. *R*_1_ = 0.0256, w*R*_2_ = 0.0592 for data with *I* ≥ 2σ(*I*), *S* (all data) = 1.030, *E*_max_, *E*_min_ = 0.48, −0.36 eÅ^−3^, respectively. CCDC 1848621.

Compound **3**, crystal data; C_14_H_21_B_10_PSe, *M* = 407.34, orthorhombic, *Pbca*, *a* = 9.9130(5), *b* = 18.9783(9), *c* = 20.7608(10) Å, *U* = 3905.8(3) Å^3^, *Z* = 8, *D*_c_ = 1.385 Mg m^−3^, μ = 1.999 mm^−1^, *F*(000) = 1632. 51443 data to θ_max_ = 31.49° were collected at 100.00(10) K on a Bruker X8 APEXII diffractometer using Mo-*K*_α_ X-radiation. A total of 6491 unique reflections (*R*_int_ = 0.0693) were used to solve and refine the structure, as for compound **1**. *R*_1_ = 0.0389, w*R*_2_ = 0.0899 for data with *I* ≥ 2σ(*I*), *S* (all data) = 1.027, *E*_max_, *E*_min_ = 0.81, −0.86 eÅ^−3^, respectively. CCDC 1848622.

Compound **4**, crystal data; C_18_H_10_F_5_PSe, *M* = 431.19, monoclinic, *P*2_1_/*c*, *a* = 16.3024(12), *b* = 7.2293(5), *c* = 14.4191(11) Å, β = 104.365(4)°, *U* = 1646.2(2) Å^3^, *Z* = 4, *D*_c_ = 1.740 Mg m^−3^, μ = 2.428 mm^−1^, *F*(000) = 848. 489984 data to θ_max_ = 34.20° were collected at 100.00(10) K on a Bruker X8 APEXII diffractometer using Mo-*K*_α_ X-radiation. A total of 6774 unique reflections (*R*_int_ = 0.0396) were used to solve and refine the structure, as for compound **1**. *R*_1_ = 0.0262, w*R*_2_ = 0.0611 for data with *I* ≥ 2σ(*I*), *S* (all data) = 1.056, *E*_max_, *E*_min_ = 0.50, −0.46 eÅ^−3^, respectively. CCDC 1848623.

For **2** and **3**, the cage C atoms were distinguished from B atoms by application of the *Vertex-Centroid Distance* (VCD) and *Boron-Hydrogen Distance* (BHD) methods [36,37,38].

## 4. Conclusions

The first example of a carborane with a catecholborolyl substituent, **1**, was prepared and fully characterized, and was shown to enhance the catalysis of a Michael addition reaction by forming an FLP with PPh_3_. A variety of carboranylphosphines were tested as FLP components in combination with B(C_6_F_5_)_3_ as catalysts of a hydrosilylation reaction, with the strongly-basic **V** performing less well than the relatively weakly basic **IVa**, whilst the very weakly basic **IX** was completely ineffective. These results demonstrate that the ability to tune the Lewis acid and/or Lewis base strength of FLP components is critical in optimizing their use as catalysts and suggest that the electronic tuneability of carborane supports offers great potential in this respect.

## Figures and Tables

**Table 1:** Results for catalysis of the Michael addition reaction in Scheme 1 by PPh_3_, the FLP **1**/PPh_3_ and **V**. ^1^

Entry	Catalyst(s)	Time (h)	Yield (%) ^2^
1	PPh_3_	6	43
2	PPh_3_	24	64
3	**1**/PPh_3_^3^	6	56
4	**1**/PPh_3_	24	76
5	**V**	6	85
6	**V**	24	92

^1^ Conditions: J. Young NMR tube; 1:1 molar ratio of **I** and **II**; 10 mol% of catalyst(s); CD_3_CN solution; room temperature. ^2^ Yield (average of two runs) determined by ^1^H NMR spectroscopy relative to mesitylene internal standard. ^3^ It was established via NMR spectroscopy that **1** and PPh_3_ did not form an adduct.

**Table 2:** Results for catalysis of the hydrosilylation reaction in Scheme 2 using the FLPs B(C_6_F_5_)_3_/PPh_2_(C_6_F_5_), B(C_6_F_5_)_3_/**IVa**, B(C_6_F_5_)_3_/**V**, and B(C_6_F_5_)_3_/**IX**. ^1^

Entry	Catalyst(s)	Time (min)	Yield (%) ^2^
1	B(C_6_F_5_)_3_/PPh_2_(C_6_F_5_)	11	89
2	B(C_6_F_5_)_3_/**IVa** ^3^	12	88
3	B(C_6_F_5_)_3_/**V** ^3^	26	80
4	B(C_6_F_5_)_3_/**IX** ^3^	-	0 ^4^

^1^ Conditions: J. Young NMR tube; 1:1 molar ratio of LA and LB components of FLP (10 mol% of each); CD_2_Cl_2_ solution; room temperature. ^2^ Yield (average of two runs) determined by ^1^H NMR spectroscopy relative to mesitylene internal standard. ^3^ It was established by NMR spectroscopy that the LA and LB components did not form an adduct. ^4^ The reagents immediately turned deep-red, indicative of the formation of oligomerized products (see Reference [23]).

## Supplementary Materials

NMR and mass spectra of all new compounds reported. Details of the catalytic runs. Crystallographic data for the structures reported in this paper have been deposited with the Cambridge Crystallographic Data Centre as supplementary publications nos. CCDC 1848620-1848623 (compounds **1**–**4**). Copies of the data can be obtained free of charge an application to CCDC, 12 Union Road, Cambridge CB2 1EZ, UK (fax: +44-1223-336033; email: deposit@ccdc.cam.ac.uk or http://www.ccdc.cam.ac.uk).
