# Supplementary material for: Exploiting the Electronic Tuneability of Carboranes as Supports for Frustrated Lewis Pairs
Source: Molecules. 2018 Nov 27;23(12):3099. doi: 10.3390/molecules23123099 (PMC6321043; doi:10.3390/molecules23123099)
Supplement: Supplementary file 1 [file molecules-23-03099-s001.pdf]

Exploiting the Electronic Tuneability of Carborans as Supports for Frustrated Lewis Pairs

Amanda Benton, Zachariah Copeland, Stephen M. Mansell, Georgina M. Rosair and Alan J. Welch

Supplementary Material

1. NMR and Mass Spectra of New Compounds

Figure S1: $^{11}B\{^{1}H\}$ NMR spectrum of compound **1** in C_6D_6

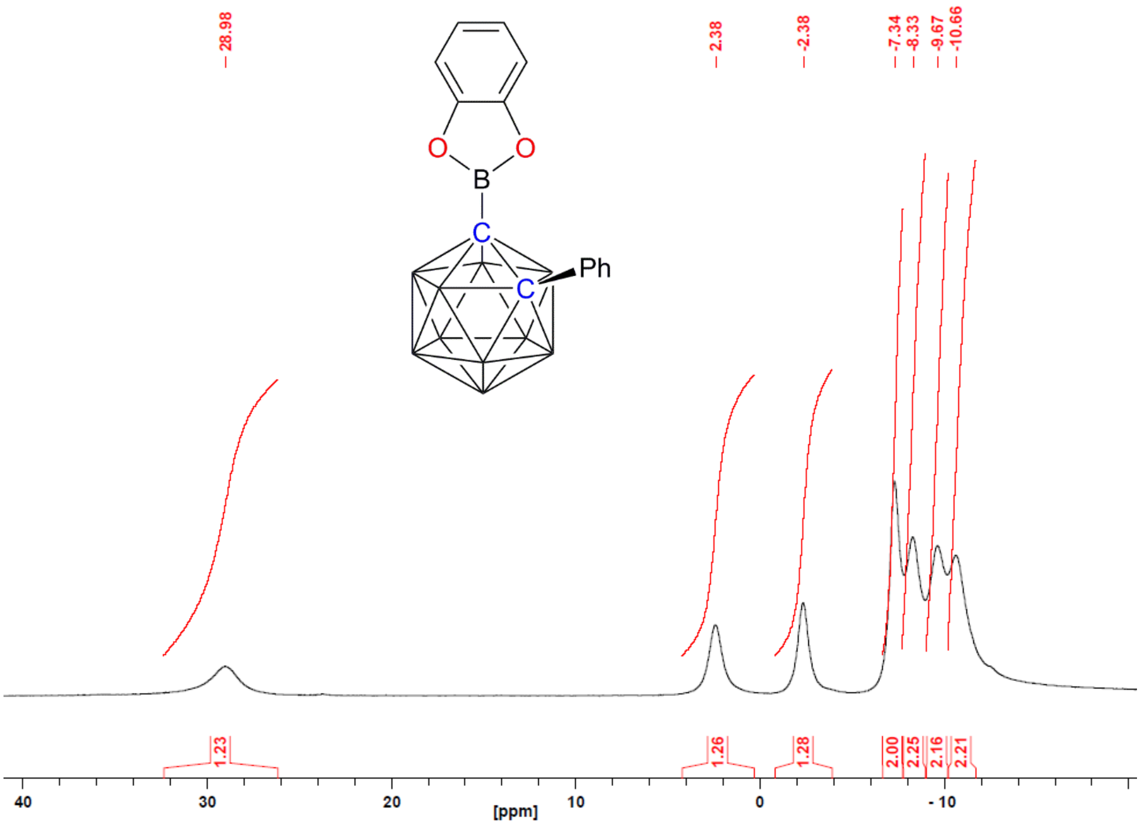

Figure S2: ^{11}B NMR spectrum of compound **1** in C_6D_6

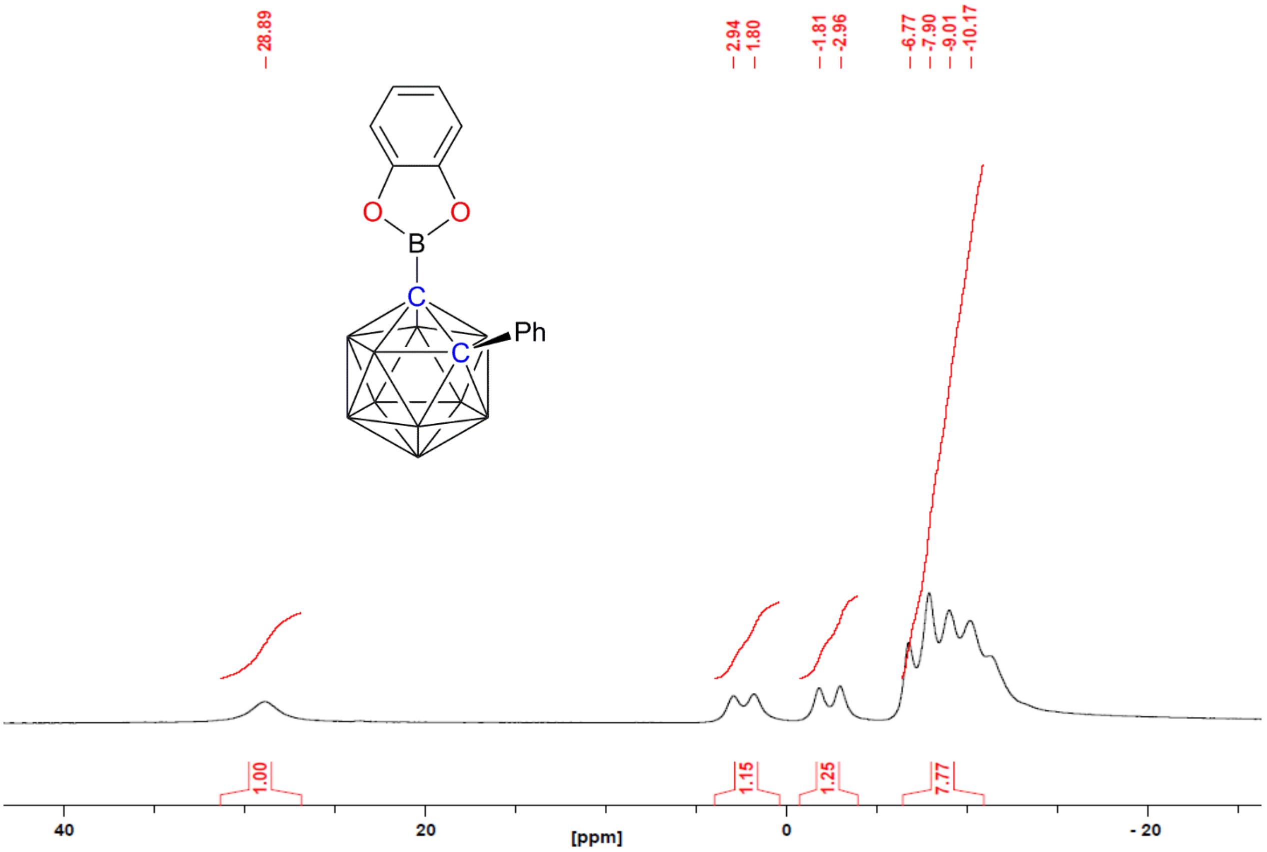

Figure S3: ^{1}H NMR spectrum (between δ 8-6 ppm) of compound **1** in C_6D_6

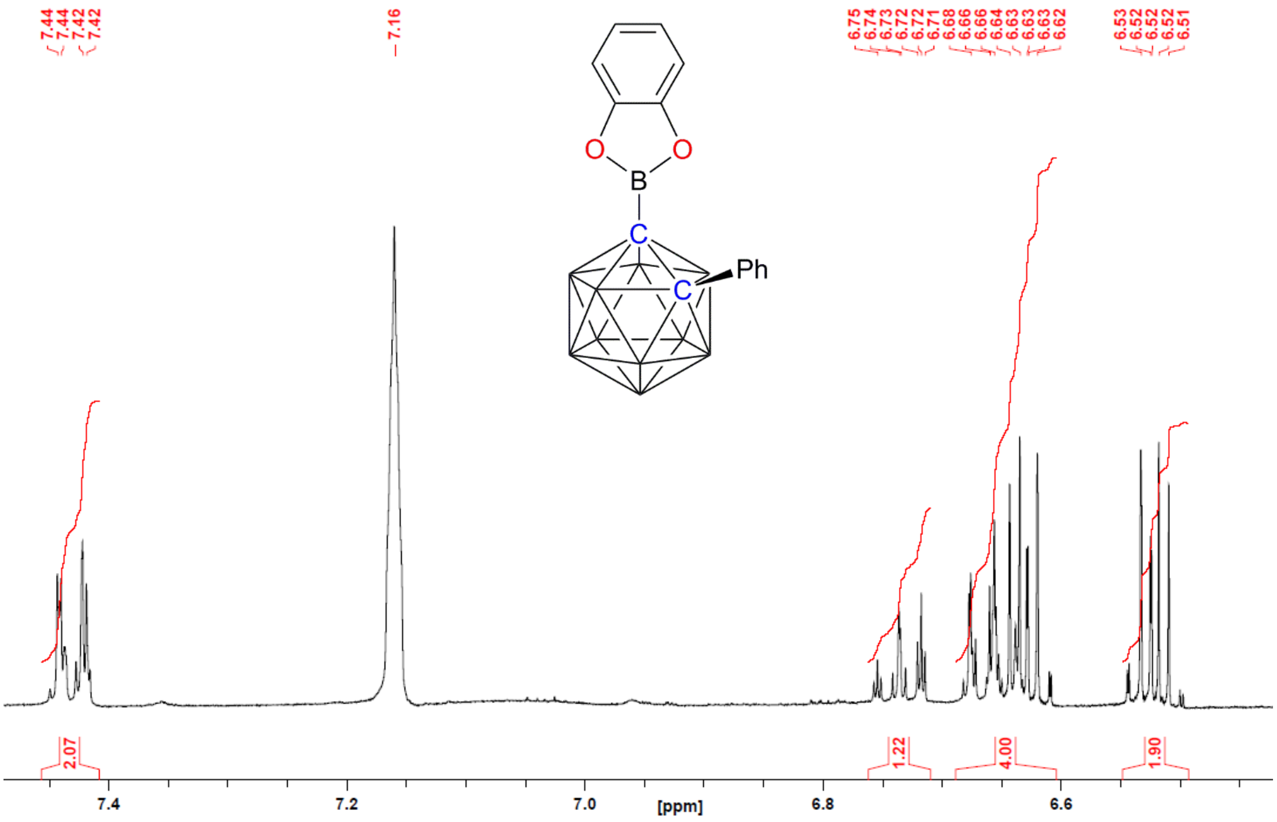

Figure S4: ^{1}H NMR spectrum (between δ 8.5-1 ppm) of compound **1** in C_6D_6

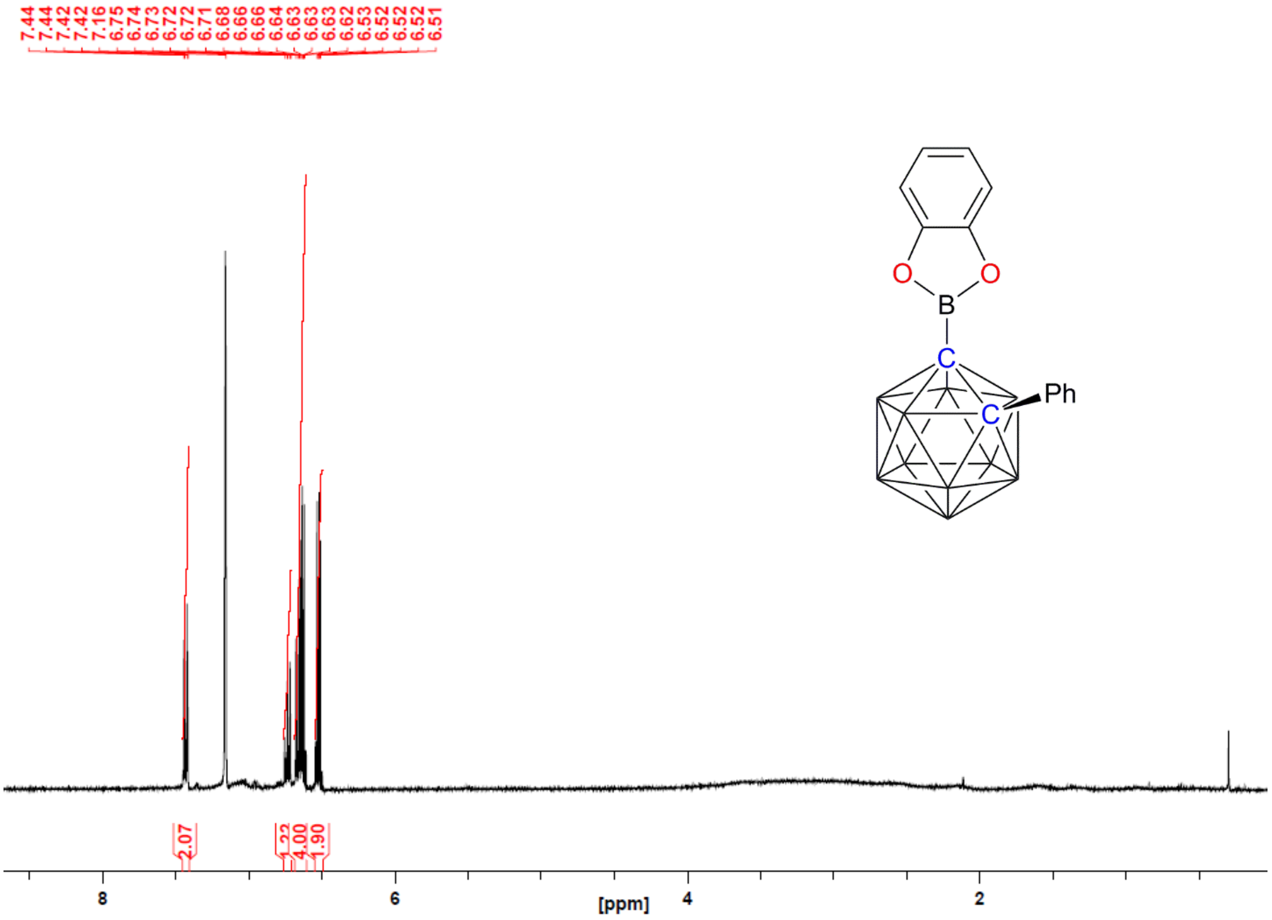

Figure S5: EI mass spectrum of compound **1**

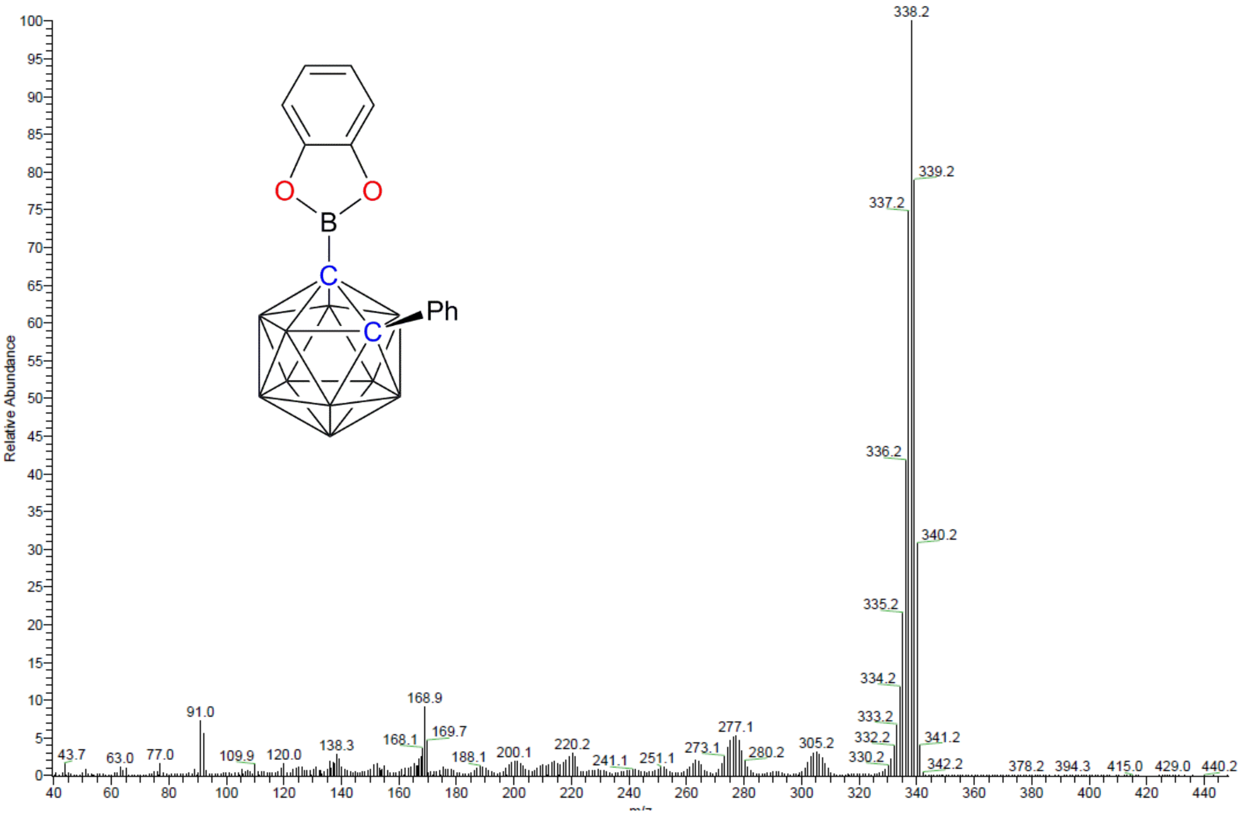

Figure S6: $^{11}B\{^{1}H\}$ NMR spectrum of compound **2** in $CDCl_3$

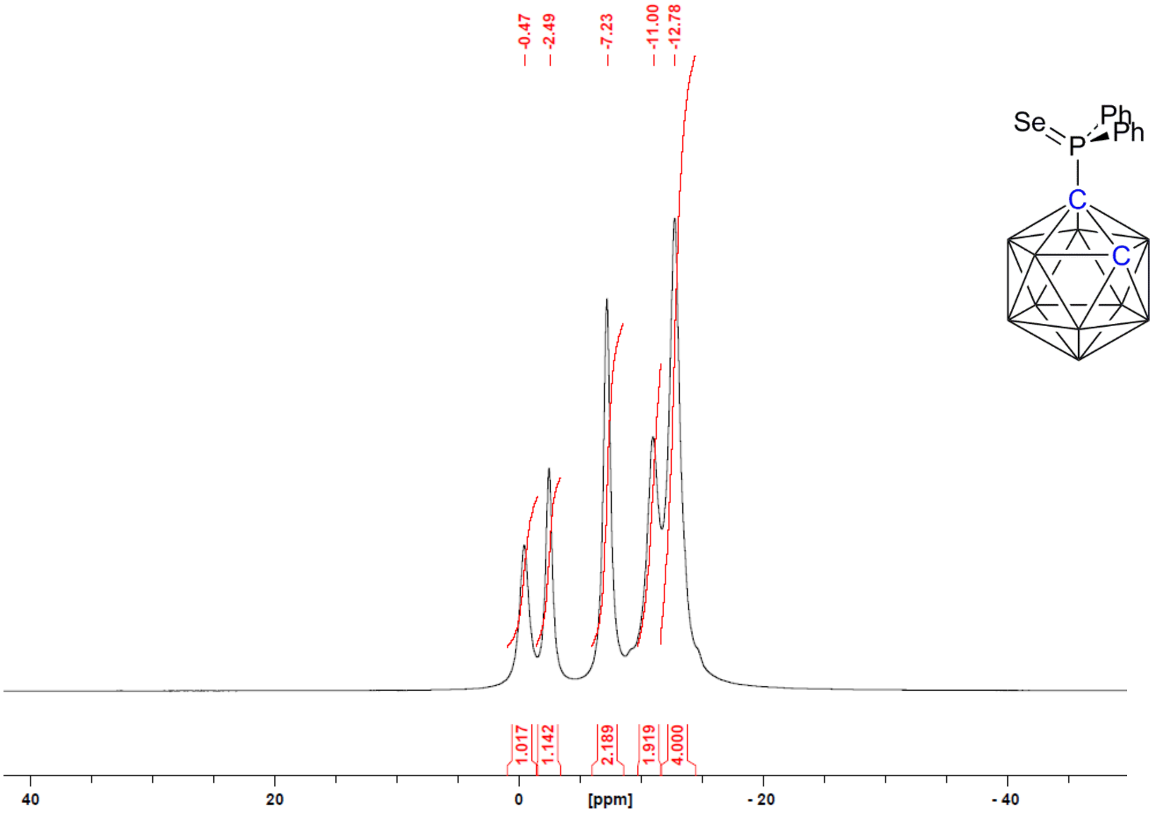

Figure S7: ^{11}B NMR spectrum of compound **2** in $CDCl_3$

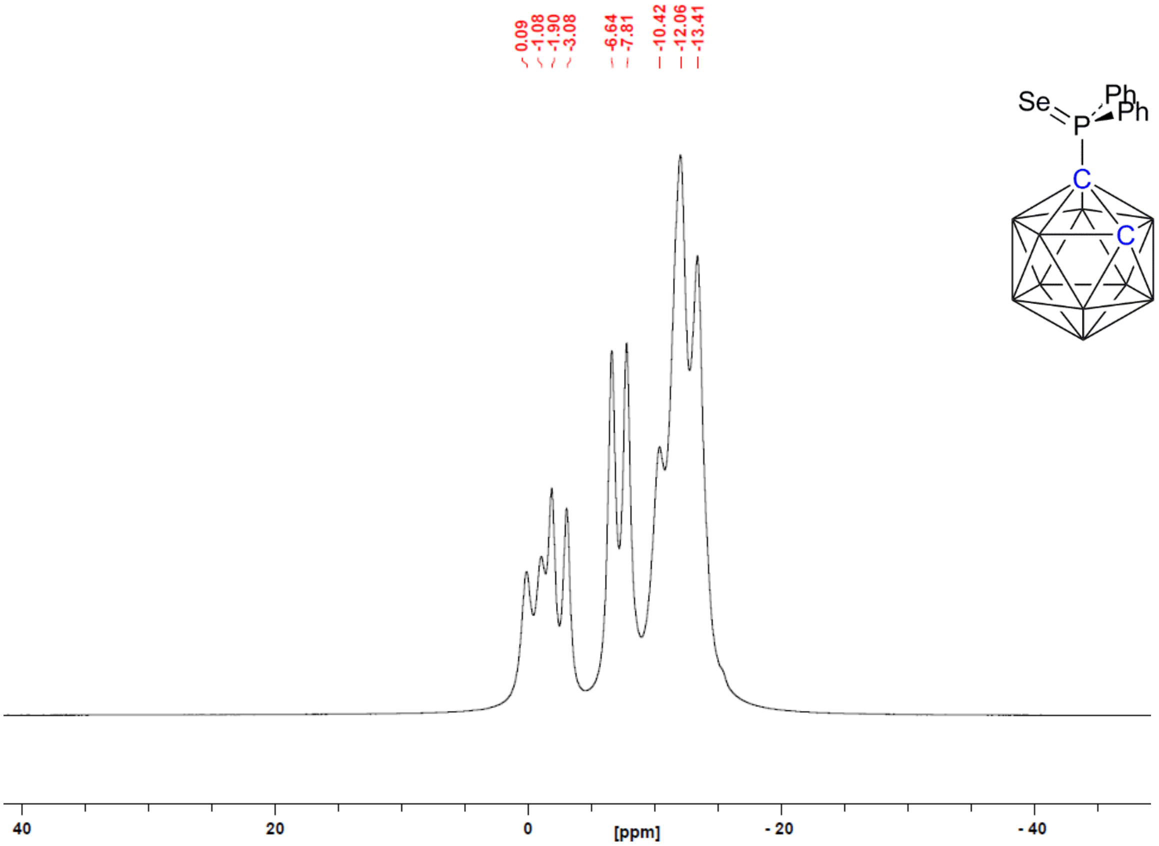

Figure S8: ^1H NMR spectrum (between δ 9-5.1 ppm) of compound **2** in $CDCl_3$

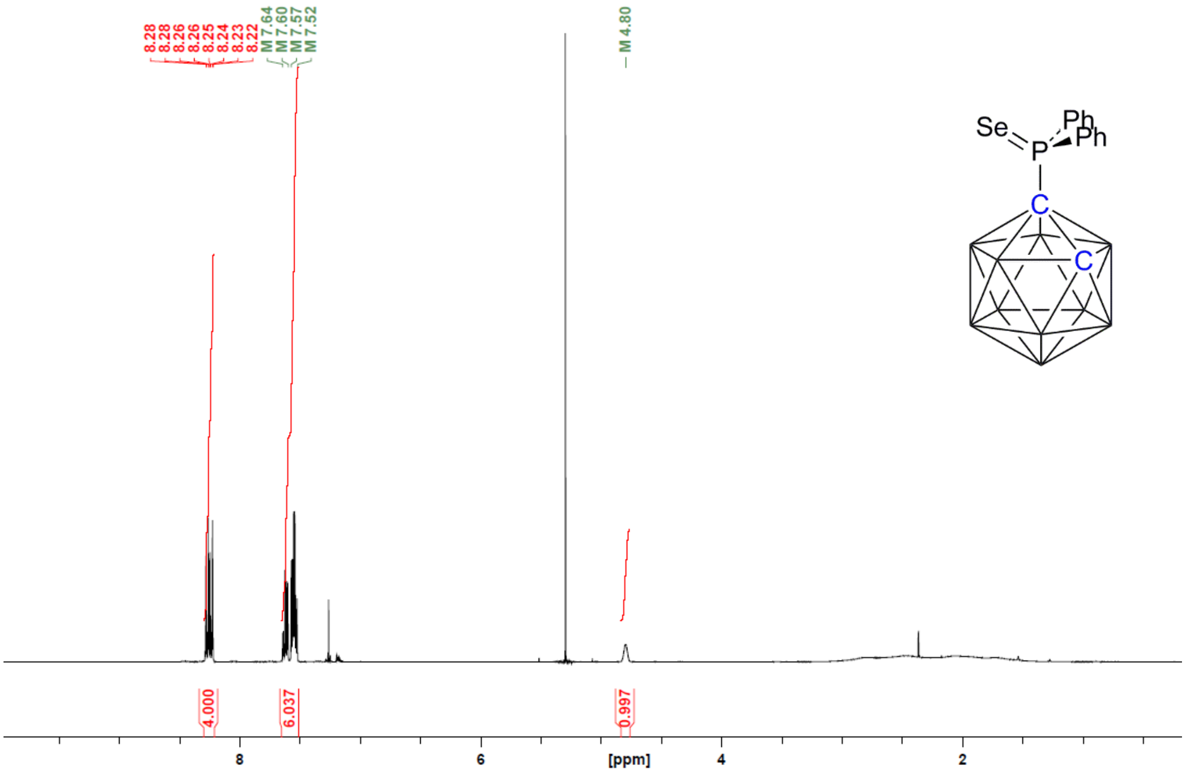

Figure S9: ^1H NMR spectrum (between δ 8.5-7.4 ppm) of compound **2** in $CDCl_3$

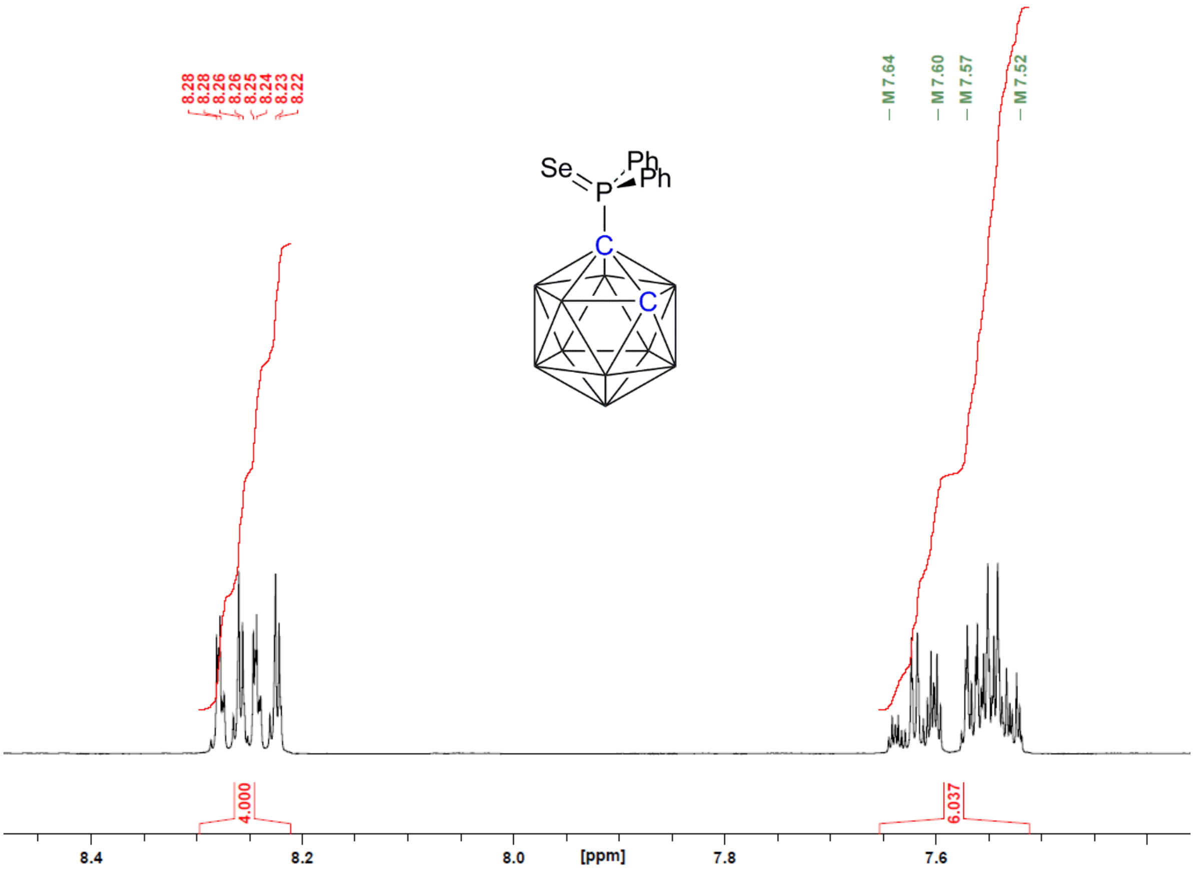

Figure S10: $^{31}P\{^{1}H\}$ NMR spectrum of compound **2** in $CDCl_3$

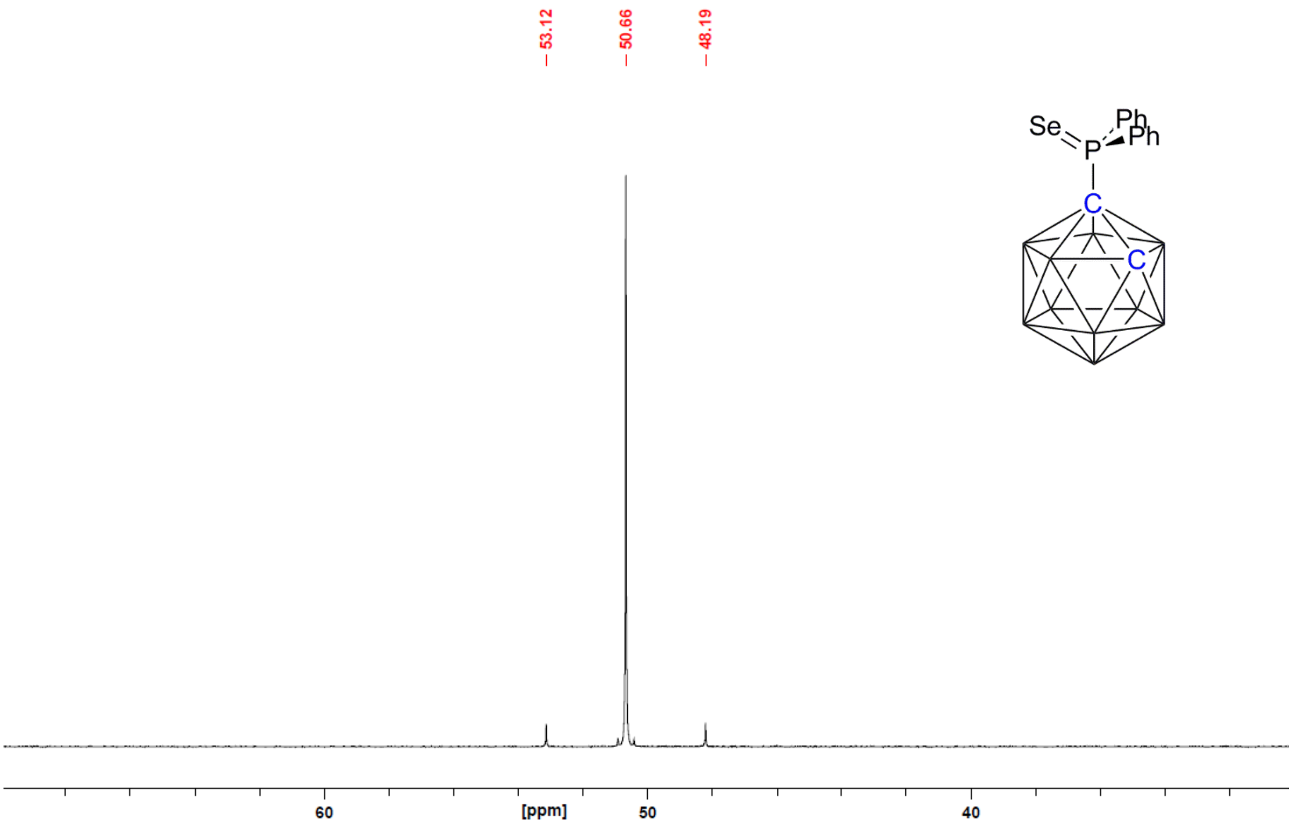

Figure S11: EI mass spectrum of compound **2**

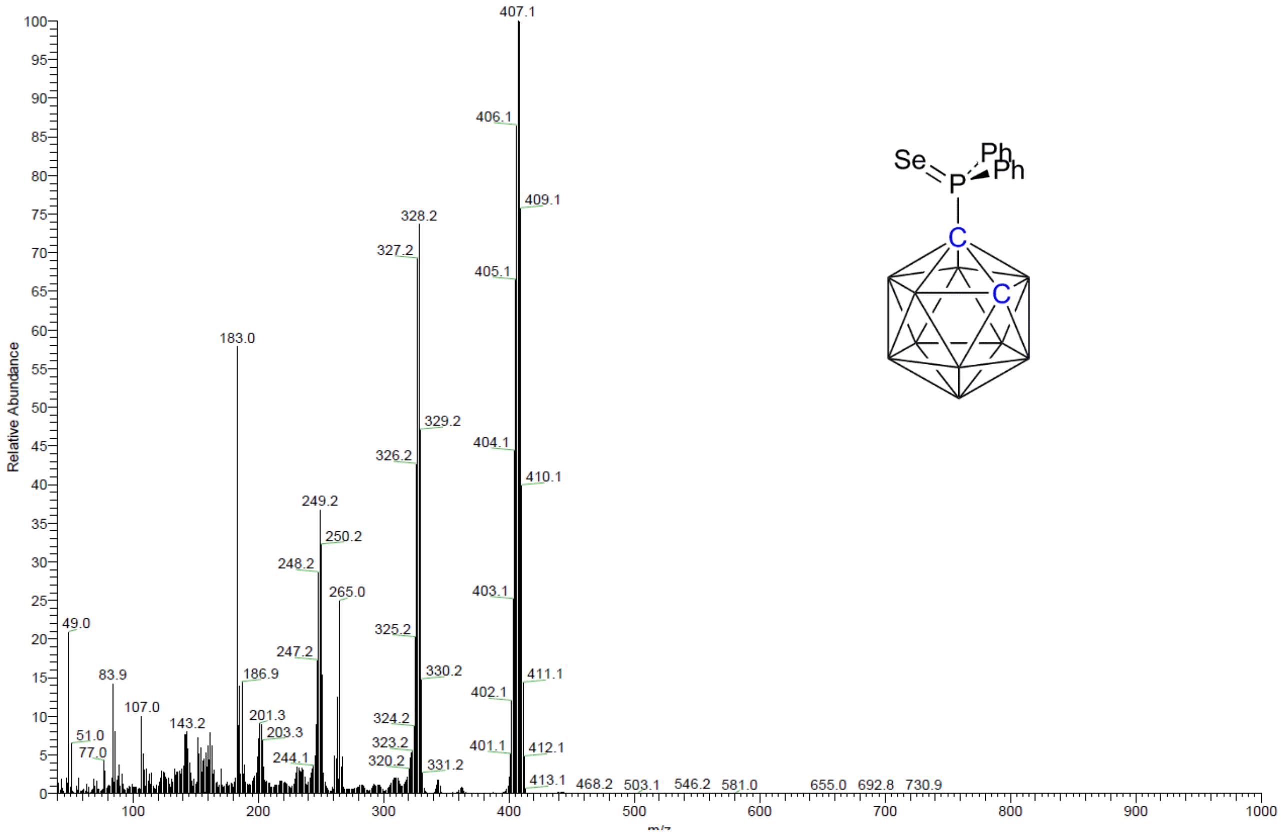

Figure S12: $^{11}B\{^{1}H\}$ NMR spectrum of compound **3** in C_6D_6

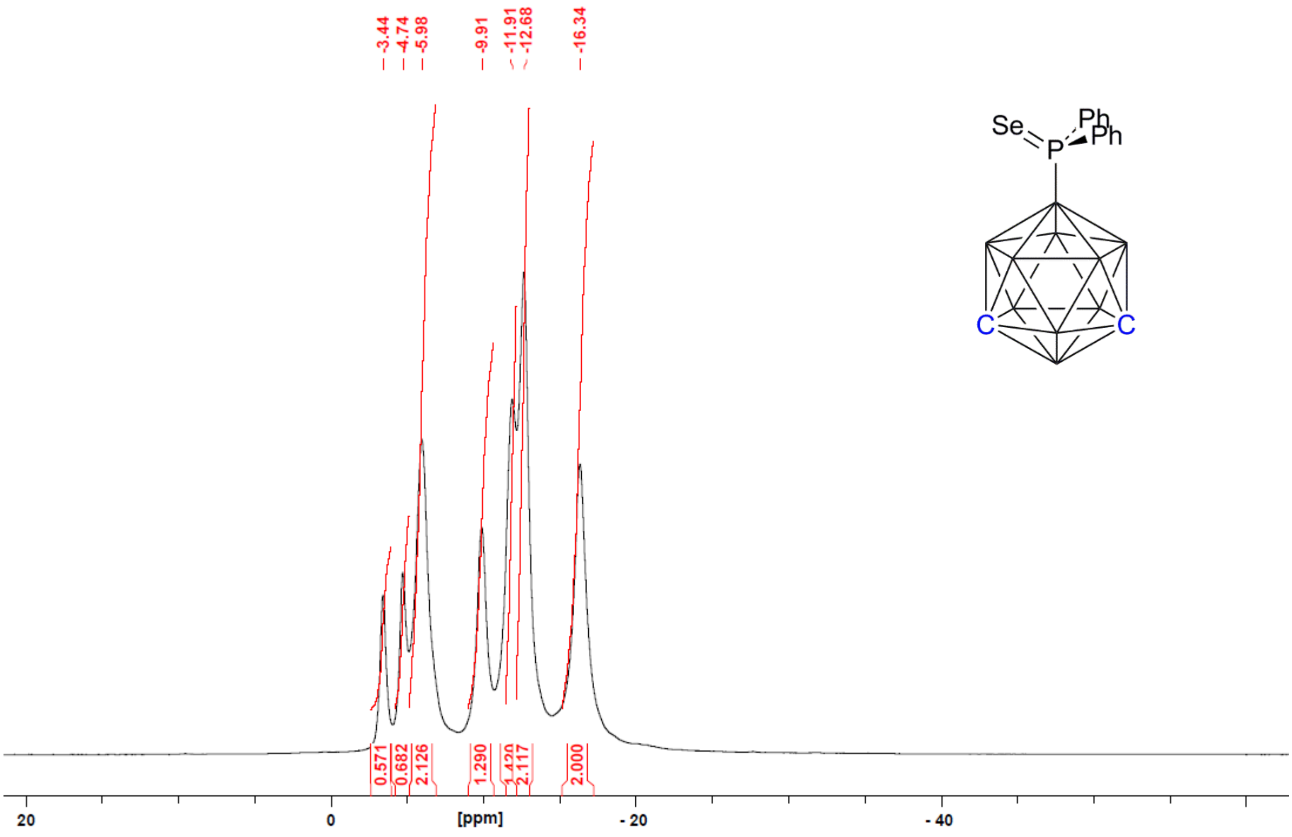

Figure S13: ^{11}B NMR spectrum of compound **3** in C_6D_6

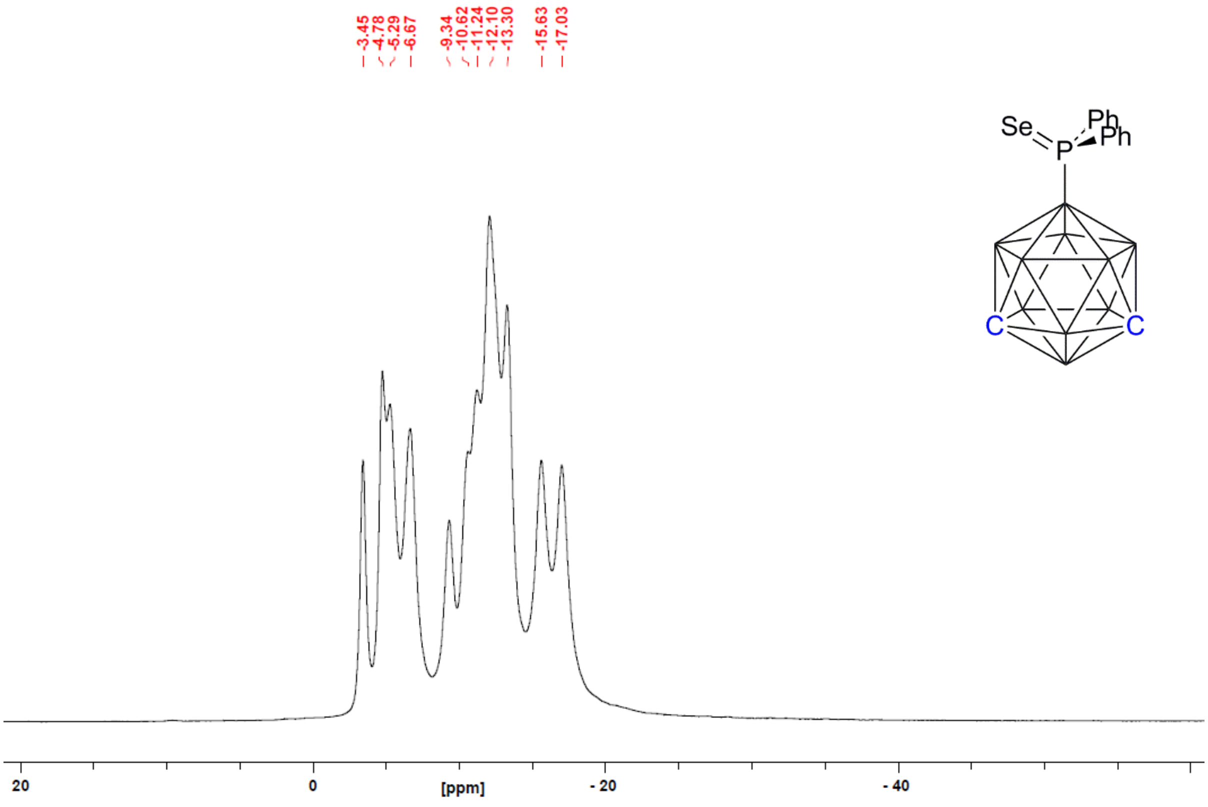

Figure S14: ^{1}H NMR spectrum (between δ 10-0.5 ppm) of compound **3** in CD_2Cl_2

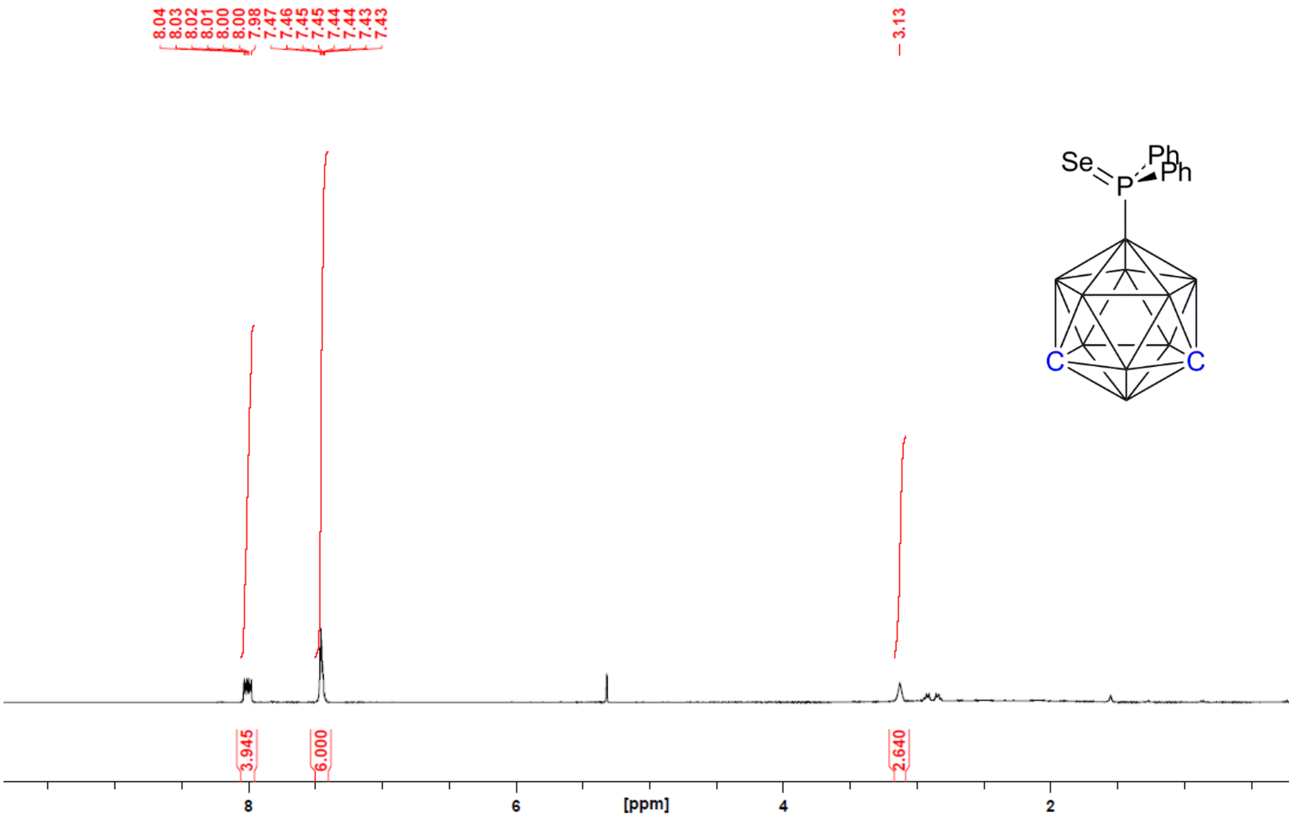

Figure S15: ^{1}H NMR spectrum (between δ 8.5-6.5 ppm) of compound **3** in CD_2Cl_2

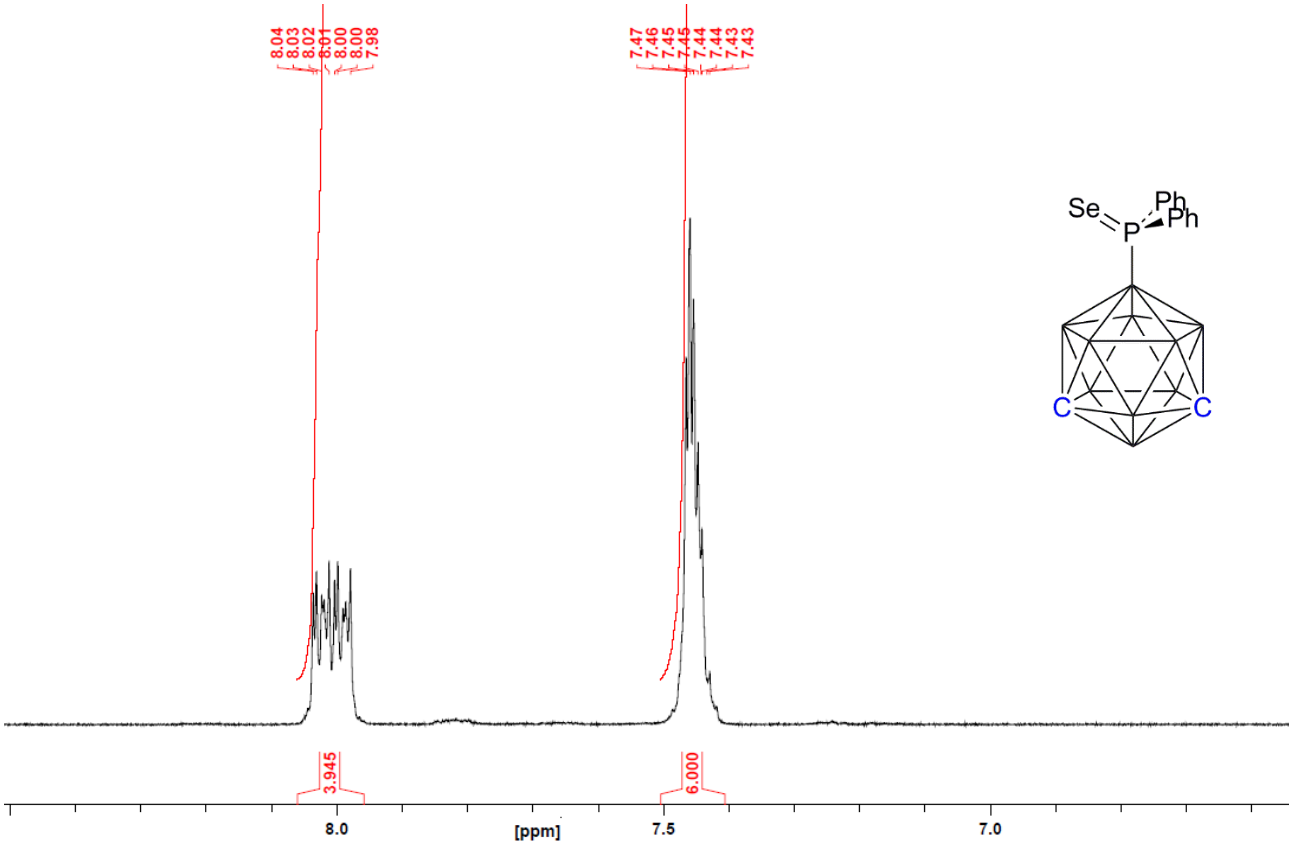

Figure S16: $^{31}P\{^1H\}$ NMR spectrum of compound **3** in C_6D_6

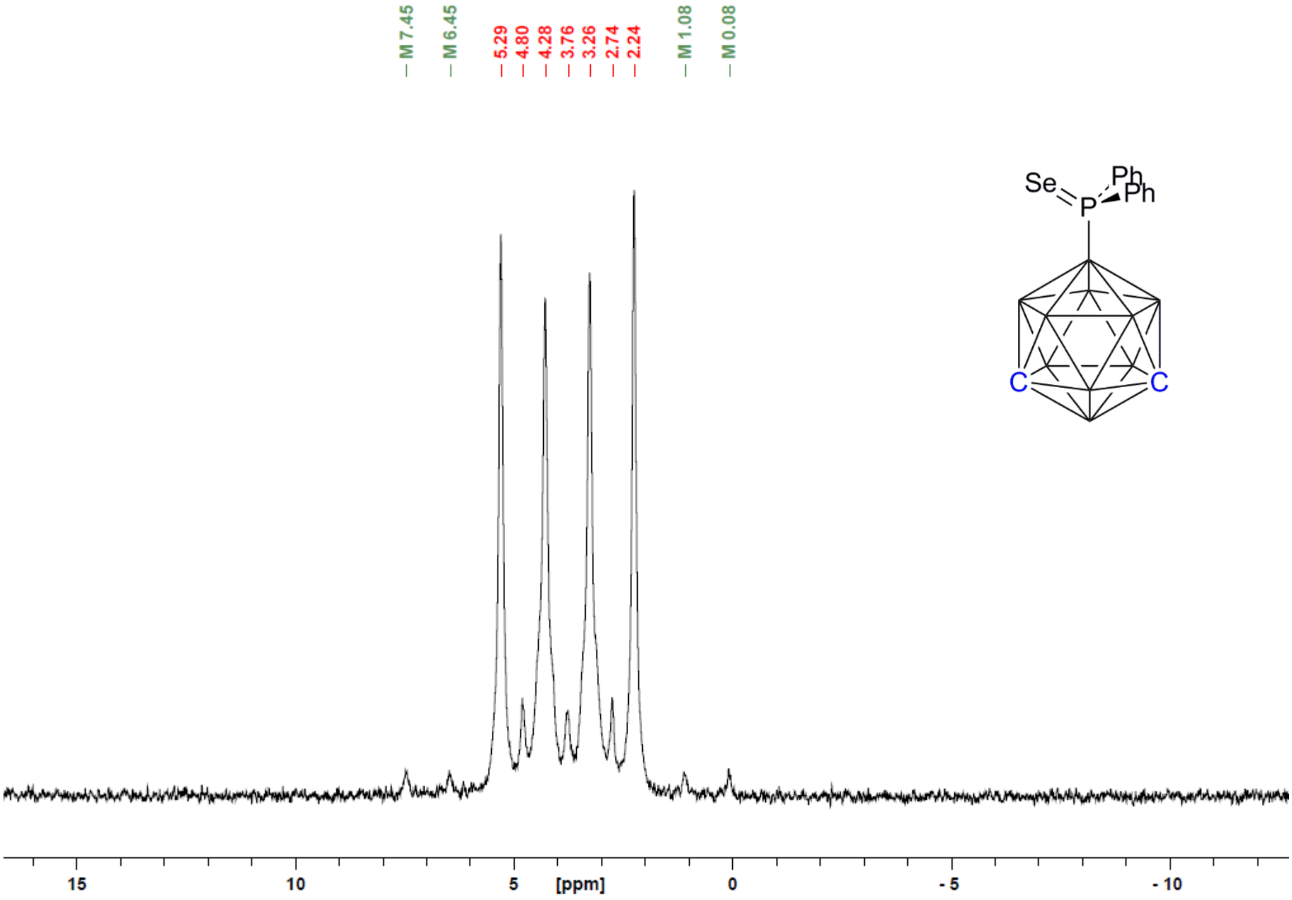

Figure S17: ^{77}Se NMR spectrum of compound **3** in C_6D_6

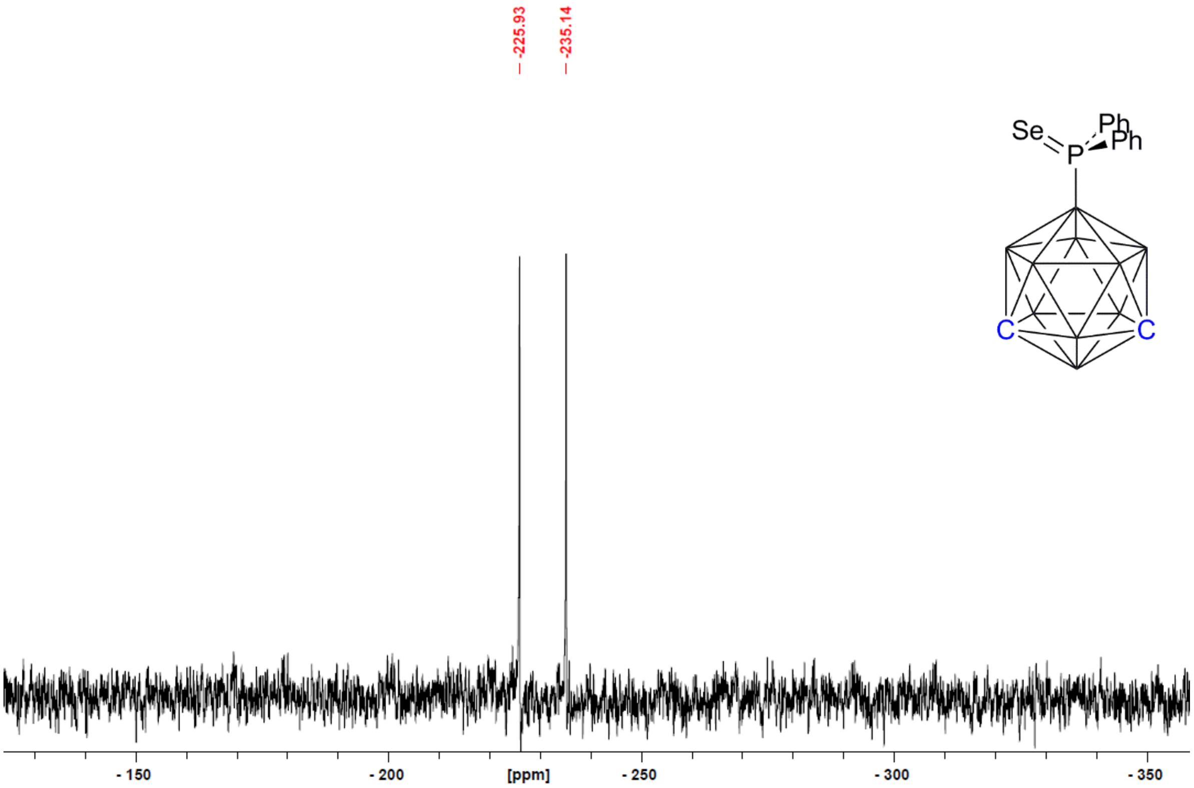

Figure S18: EI mass spectrum of compound **3**

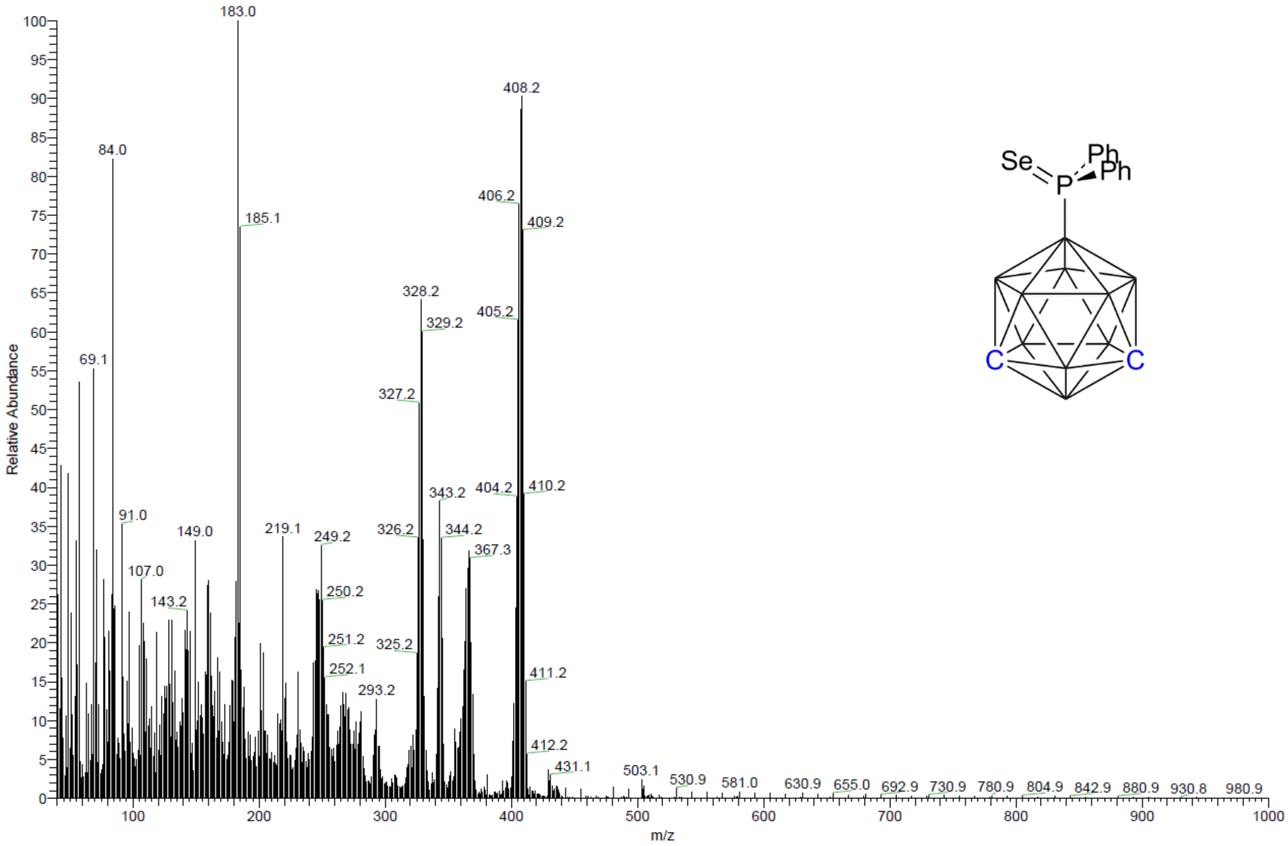

Figure S19: ^{1}H NMR spectrum (between δ 9.5-1 ppm) of compound **4** in $CDCl_3$

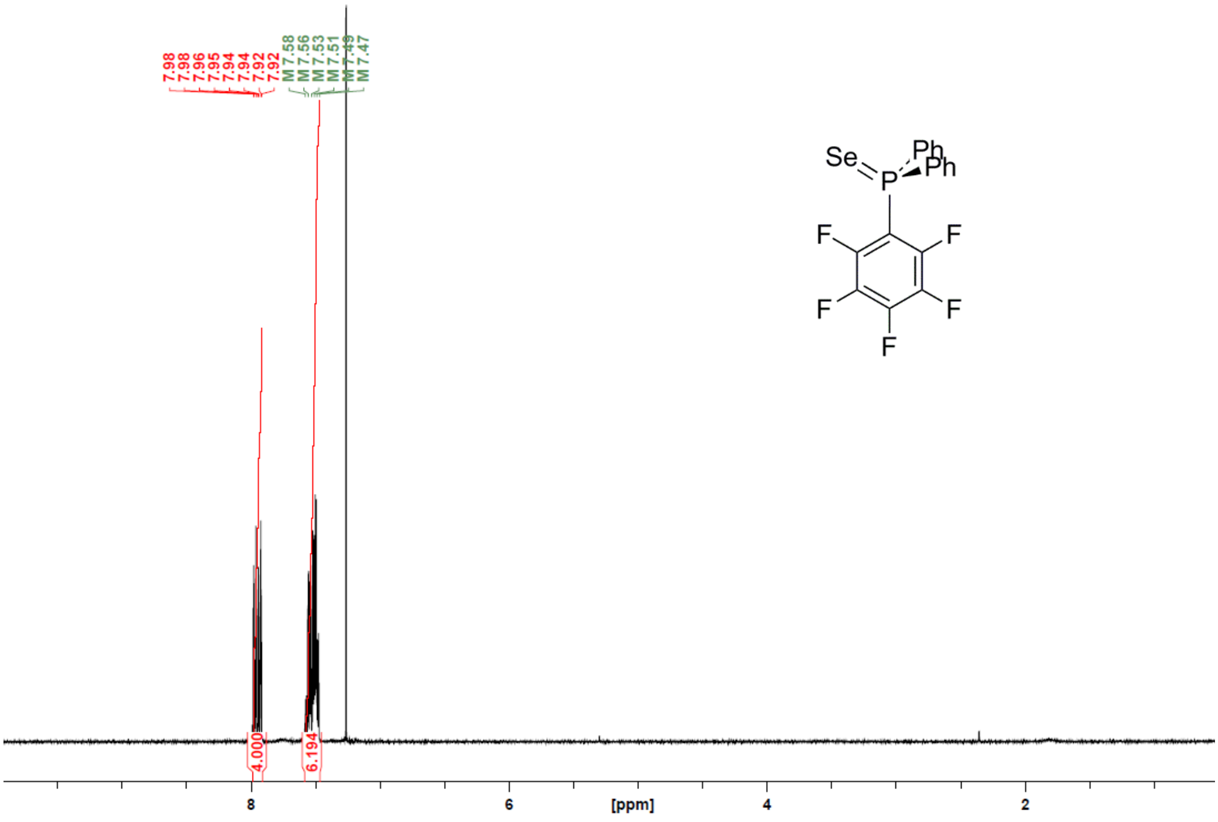

Figure S20: ^{1}H NMR spectrum (between δ 8.5-6.9 ppm) of compound **4** in $CDCl_3$

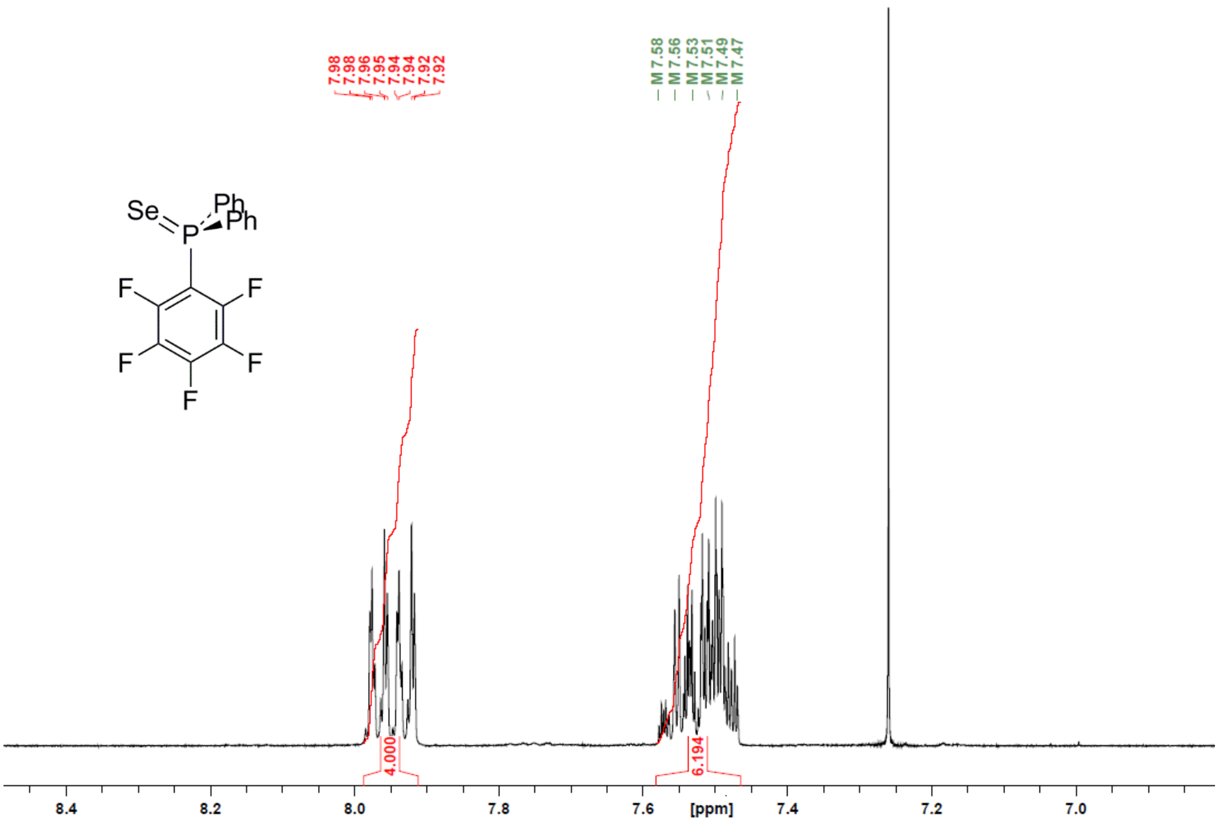

Figure S21: $^{31}P\{^{1}H\}$ NMR spectrum of compound **4** in $CDCl_3$

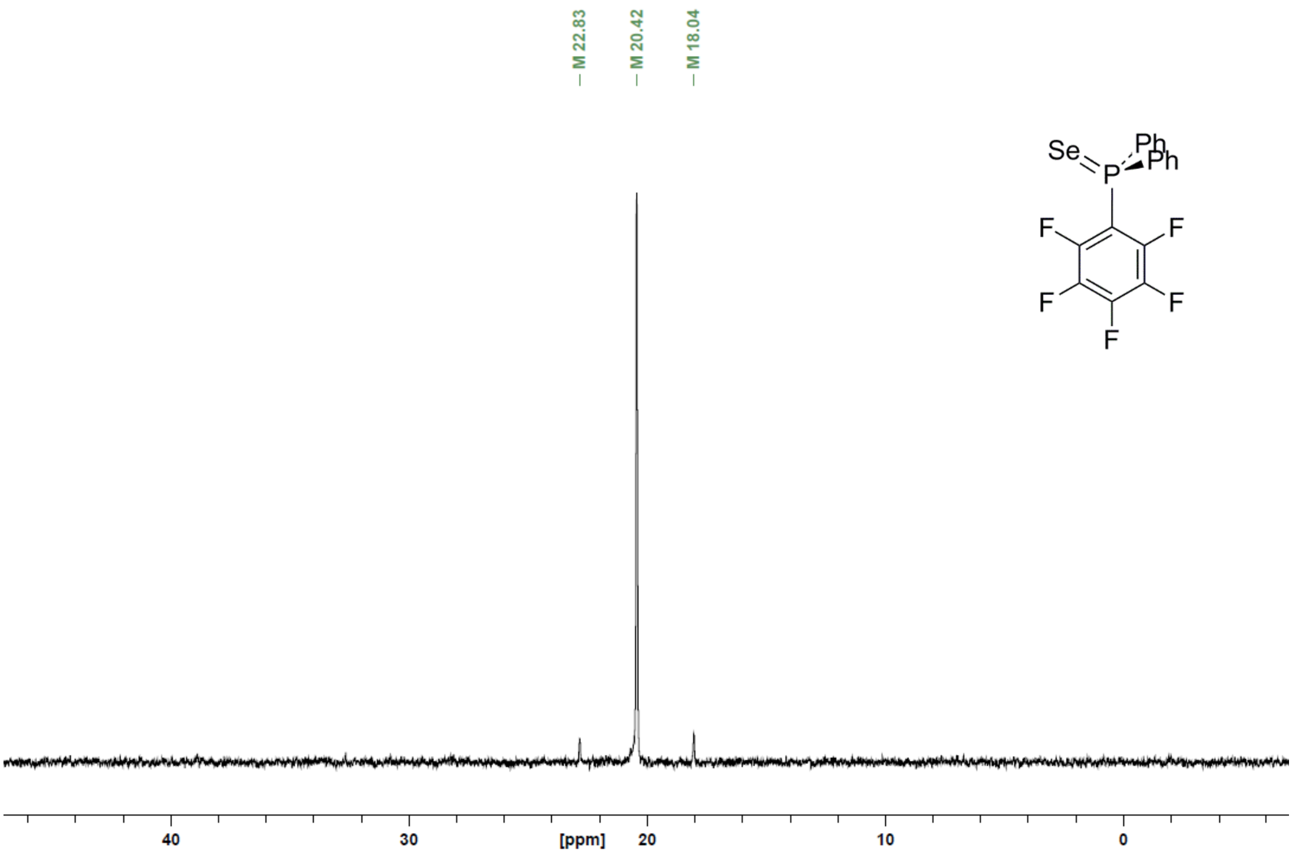

Figure S22: ^{19}F NMR spectrum of compound **4** in $CDCl_3$

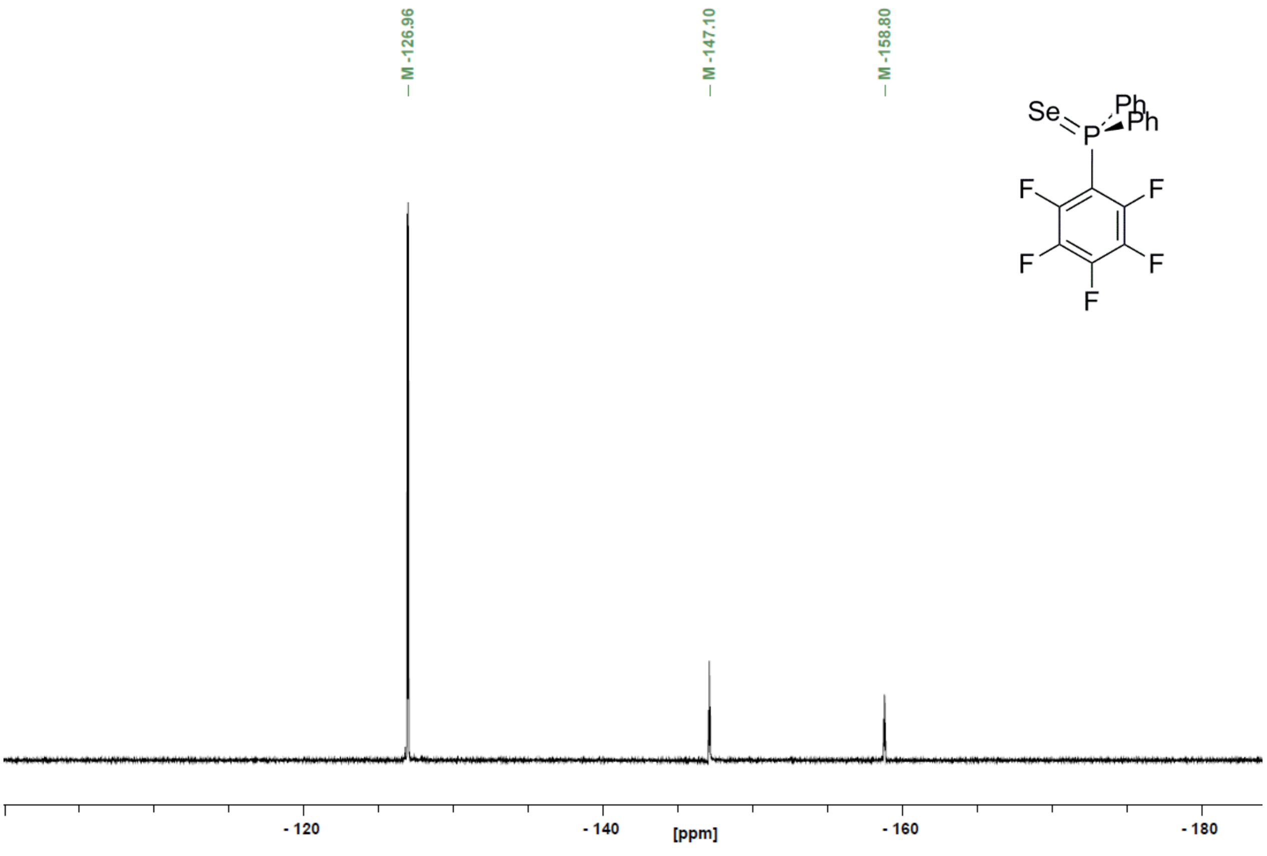

Figure S23: EI mass spectrum of compound **4**

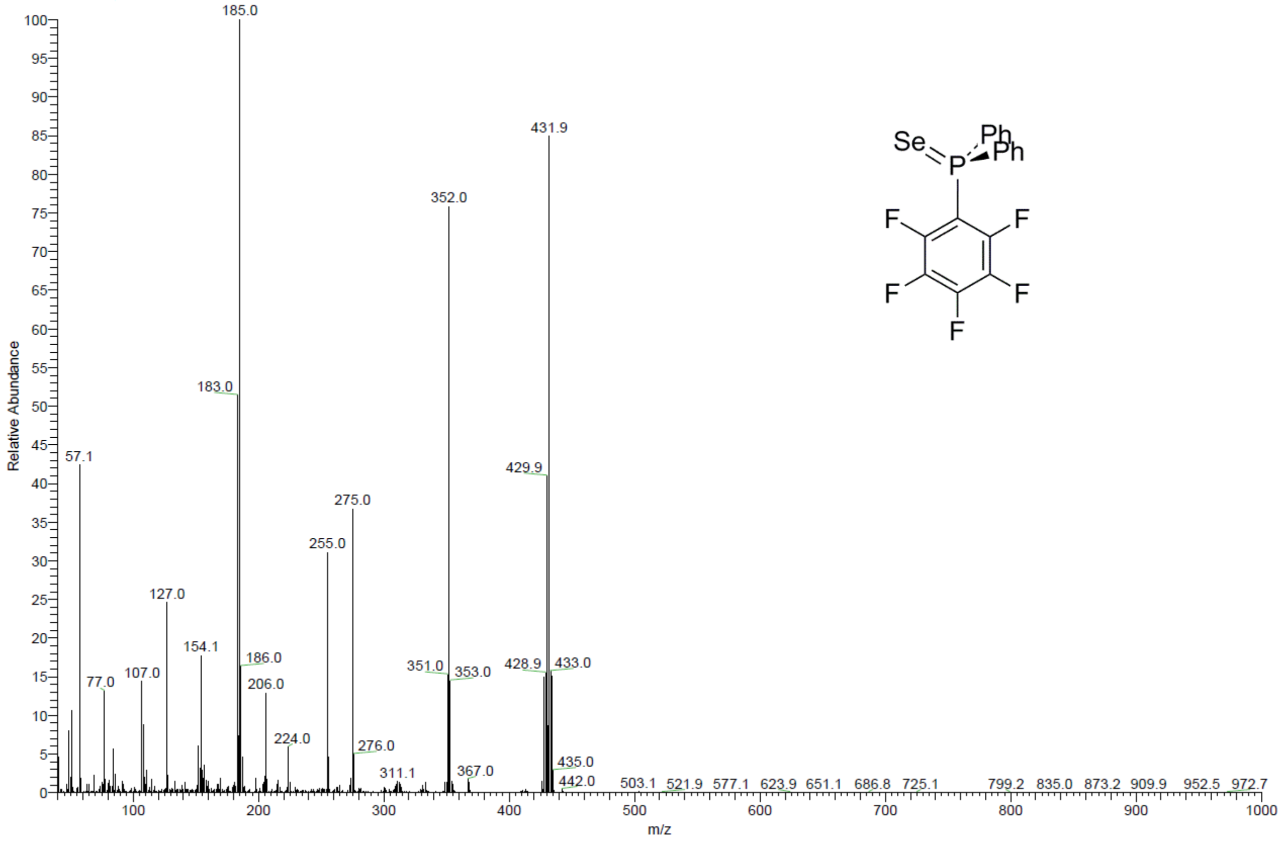

2. Catalytic Studies

2.1 Michael Addition Reaction

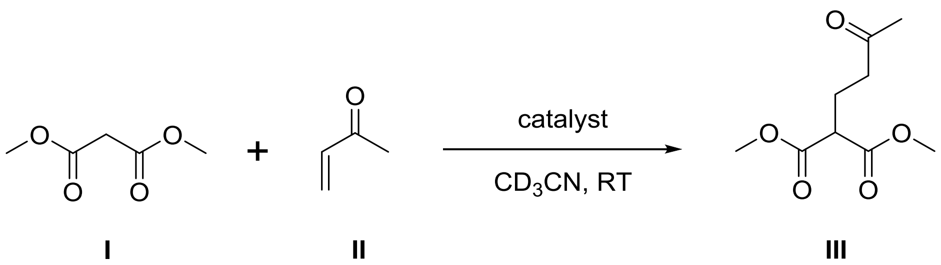

The catalyst (0.04 mmol) was placed in a J. Young NMR tube followed by, in order, dried and degassed CD_3CN (0.7 mL), dimethylmalonate (**I**, 0.4 mmol) and methylvinylketone (**II**, 0.4 mmol). Finally, mesitylene as internal standard (0.2 mmol) was added and the tube sealed and shaken. The reaction was monitored by ^1H NMR spectroscopy every hour, recording the integral of the resonance at δ 2.51 ppm (2H) of the product dimethyl-2-(3-oxybutyl)malonate (**III**) relative to the integral of the resonance at δ 6.80 ppm (3H) of mesitylene. Results below are the average of two runs.

2.1.1 PPh_3 catalyst

Hours	Product Integral	% Yield
0.68	0.75	18.8
1.72	1.14	28.6
2.42	1.33	33.3
3.33	1.50	37.4
4.28	1.64	40.9
5.23	1.72	43.1
6.46	1.87	46.8
23.65	2.57	64.3

*2.1.2 **1**/PPh_3 catalyst*

Hours	Product Integral	% Yield
0.35	0.70	17.6
1.00	0.85	21.3
2.83	1.63	40.8
4.03	1.85	46.2
5.07	2.02	50.6
6.02	2.25	56.1
23.55	3.03	75.9

*2.1.3 **V** catalyst*

Hours	Product Integral	% Yield
0.12	2.06	50.2
1.00	3.10	77.6
2.13	3.21	80.2
2.98	3.25	81.3
4.00	3.31	82.7
5.08	3.348	83.7
6.10	3.400	85.0
23.90	3.696	92.4

2.1 Hydrosilylation Reaction

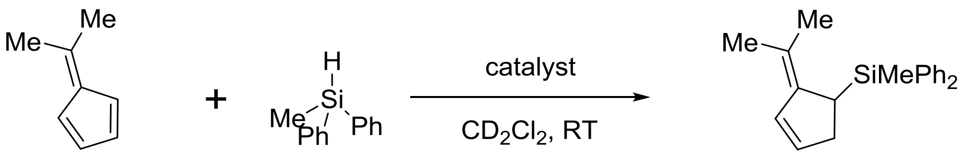

VI **VII** **VIII**

The Lewis base component of the FLP catalyst (0.04 mmol) was placed in a J. Young NMR tube followed by, in order, 6,6-dimethylfulvene (**VI**, 0.4 mmol), methyldiphenylsilane (**VII**, 0.4 mmol) and dry and degassed CD_2Cl_2 (0.7 mL). Finally, in the glovebox, the Lewis acid component [$B(C_6F_5)_3$, 0.04 mmol] was added and the tube sealed and shaken. The reaction was monitored by ^1H NMR spectroscopy as soon as possible (this reaction is much quicker than the Michael addition reaction), recording the integral of the resonance at δ 6.51 ppm (1H) of the product **VIII** relative to the integral of the resonance at δ 2.35 ppm (9H) of mesitylene. In the case of **IX** the contents of the NMR tube turned immediately deep-red on addition of the Lewis acid, suggesting oligomerization, and no product was detected. Results below are the average of two runs.

Lewis base	Time (/mins)	% Yield
$PPh_2(C_6F_5)$	11	89
**IVa**	12	88
**V**	26	80
**IX**	-	0
